# Optimising Controlled Human Malaria Infection Studies Using Cryopreserved *P. falciparum* Parasites Administered by Needle and Syringe

**DOI:** 10.1371/journal.pone.0065960

**Published:** 2013-06-18

**Authors:** Susanne H. Sheehy, Alexandra J. Spencer, Alexander D. Douglas, B. Kim Lee Sim, Rhea J. Longley, Nick J. Edwards, Ian D. Poulton, Domtila Kimani, Andrew R. Williams, Nicholas A. Anagnostou, Rachel Roberts, Simon Kerridge, Merryn Voysey, Eric R. James, Peter F. Billingsley, Anusha Gunasekera, Alison M. Lawrie, Stephen L. Hoffman, Adrian V. S. Hill

**Affiliations:** 1 Centre for Clinical Vaccinology and Tropical Medicine, University of Oxford, Oxford, United Kingdom; 2 The Jenner Institute Laboratories, University of Oxford, Oxford, United Kingdom; 3 Sanaria Inc., Rockville, Maryland, United States of America; 4 Centre for Geographical Medical Research (Coast), Kenya Medical Research Institute, Kilifi, Kenya; 5 Centre for Statistics in Medicine, University of Oxford, Oxford, United Kingdom; Aeras, United States of America

## Abstract

**Background:**

Controlled human malaria infection (CHMI) studies have become a routine tool to evaluate efficacy of candidate anti-malarial drugs and vaccines. To date, CHMI trials have mostly been conducted using the bite of infected mosquitoes, restricting the number of trial sites that can perform CHMI studies. Aseptic, cryopreserved *P. falciparum* sporozoites (PfSPZ Challenge) provide a potentially more accurate, reproducible and practical alternative, allowing a known number of sporozoites to be administered simply by injection.

**Methodology:**

We sought to assess the infectivity of PfSPZ Challenge administered in different dosing regimens to malaria-naive healthy adults (n = 18). Six participants received 2,500 sporozoites intradermally (ID), six received 2,500 sporozoites intramuscularly (IM) and six received 25,000 sporozoites IM.

**Findings:**

Five out of six participants receiving 2,500 sporozoites ID, 3/6 participants receiving 2,500 sporozoites IM and 6/6 participants receiving 25,000 sporozoites IM were successfully infected. The median time to diagnosis was 13.2, 17.8 and 12.7 days for 2,500 sporozoites ID, 2,500 sporozoites IM and 25,000 sporozoites IM respectively (Kaplan Meier method; *p = 0.024* log rank test).

**Conclusions:**

2,500 sporozoites ID and 25,000 sporozoites IM have similar infectivities. Given the dose response in infectivity seen with IM administration, further work should evaluate increasing doses of PfSPZ Challenge IM to identify a dosing regimen that reliably infects 100% of participants.

**Trial Registration:**

ClinicalTrials.gov NCT01465048

## Introduction

The deliberate infection of human participants with micro-organisms (challenge studies) have contributed uniquely to our understanding of the pathogenesis, immune responses, treatment and prevention of numerous microbial diseases. [1] *Plasmodium falciparum* malaria is a microbe particularly suited to challenge studies as it has a relatively short and predictable asymptomatic period, a well-established diagnostic laboratory test (thick film microscopy), and no known long-term sequelae or infectious state following appropriate treatment. Studies involving controlled human malaria infection (CHMI) have become established as a key tool to assess the efficacy of malaria vaccine and drug candidates, allowing unprecedented detailed evaluation of parasite growth and immunological responses. [2] Since the late 1980s, the number of institutions performing CHMI studies with *P. falciparum* has been growing and a total of 1,343 participants were experimentally infected with *P. falciparum* between 1985 and 2009. [3] With an increasing number of candidate malaria vaccines being developed, the number of centres conducting CHMI is set to expand to increase the testing capacity worldwide.

The majority of CHMI trials to date have been performed using the NF54 stain of *P. falciparum* [4], [5] or 3D7 (which is a clone of NF54) [6] sporozoites delivered by mosquito bite. [2] Standardisation of this method over the last 20 years has established a protocol that reliably infects 100% of malaria-naïve individuals with rare exceptions, providing a stringent, widely accepted *in vivo* efficacy assessment of novel drugs and vaccines. [2] Whilst the model has the benefit of mimicking the natural route of infection, it is limited by the inability to predefine and control the number of sporozoites inoculated, meaning this number can vary by several thousand sporozoites. [7]–[11] Mosquito bite CHMI studies can only be performed in centres with access to an appropriate insectary and entomology staff. This restriction considerably limits the number of sites that can perform such studies and has provided a major obstacle to the conduct of CHMI trials in malaria endemic regions.

In principle, the most accurate and practical way of dosing sporozoites is to inject directly by needle and syringe. [2] As well as the practical advantages of ease of administration and ability to ‘challenge’ participants over an extended period rather than the same day, this method would reduce variation in infectious dose between parallel clinical trials at multiple sites or sequential clinical trials at the same site.

Sanaria Inc. is a biotechnology company that has developed infectious, aseptic, purified, cryopreserved *P. falciparum* sporozoites (NF54), which can be administered by needle and syringe. [12] The salivary glands of aseptic *A. stephensi* mosquitoes infected with *P. falciparum* sporozoites are removed by dissection and triturated to release the sporozoites which are purified, counted and cryopreserved at a specified concentration to produce the challenge inoculum; PfSPZ Challenge. The first CHMI trial using PfSPZ Challenge was performed in 2010. [12] In this dose escalation study, three doses of PfSPZ Challenge (2,500, 10,000 and 25,000 sporozoites) administered intradermally (ID) each successfully infected only 5 out of 6 injected participants (83%). If PfSPZ Challenge is to provide an alternative to the mosquito bite CHMI, an administration regimen must be identified that reliably infects 100% of participants.

Murine data have shown that the route of administration of cryopreserved sporozoites is a key determinant of infectivity. C57/BL6 mice (n = 5 per group) were injected with 50,000 sporozoites administered by intravenous (IV), IM, subcutaneous (SC) or ID routes and subsequent parasite liver load determined by *in vivo* imaging. Results clearly demonstrated that IV administration was associated with the greatest infectivity. However, IM administration was the next most effective route of delivery, associated with greater infectivity than SC or ID routes of injection. [13] Given the potential practical limitations of an IV CHMI model and promising results already seen with ID administration of PfSPZ Challenge, we sought to conduct a trial to assess the infectivity of PfSPZ Challenge administered IM with a view to establishing a dosing regimen that reliably infects 100% of participants.

The proposed trial provided a unique opportunity to examine immunological responses induced in malarial naïve volunteers following the first episode of *P. falciparum* infection. Data from murine and clinical studies have suggested a critical role of IFN-γ producing T cells in reducing the liver burden of *P. falciparum* infection. [14]–[20]. However, infection has only been associated with modest responses to well known pre-erythrocytic antigens (such as Thrombospondin Related Adhesion Protein (TRAP), Circumsporozoite Surface Protein (CSP), Cell-Traversal protein for Ookinetes and Sporozoites (CelTOS), Liver Stage Antigen 1 (LSA1), Exported Protein 1 (EXP1) and Merozoite Surface Protein (MSP) [21]–[27] which have never been correlated with protection. We sought to assess T-cell responses to other novel pre-erythrocytic antigens in the hope of identifying new antigenic targets for future vaccine development.

## Methods

The protocol for this trial and supporting CONSORT checklist are available as supporting information; see Checklist S1 and Protocol S1.

### Objective

The objective of the study was to evaluate the infectivity of varying doses and routes of administration of aseptic cryopreserved *P. falciparum* sporozoites (PfSPZ Challenge) administered by needle and syringe.

### Study Design & Participants

**Figure 1:**
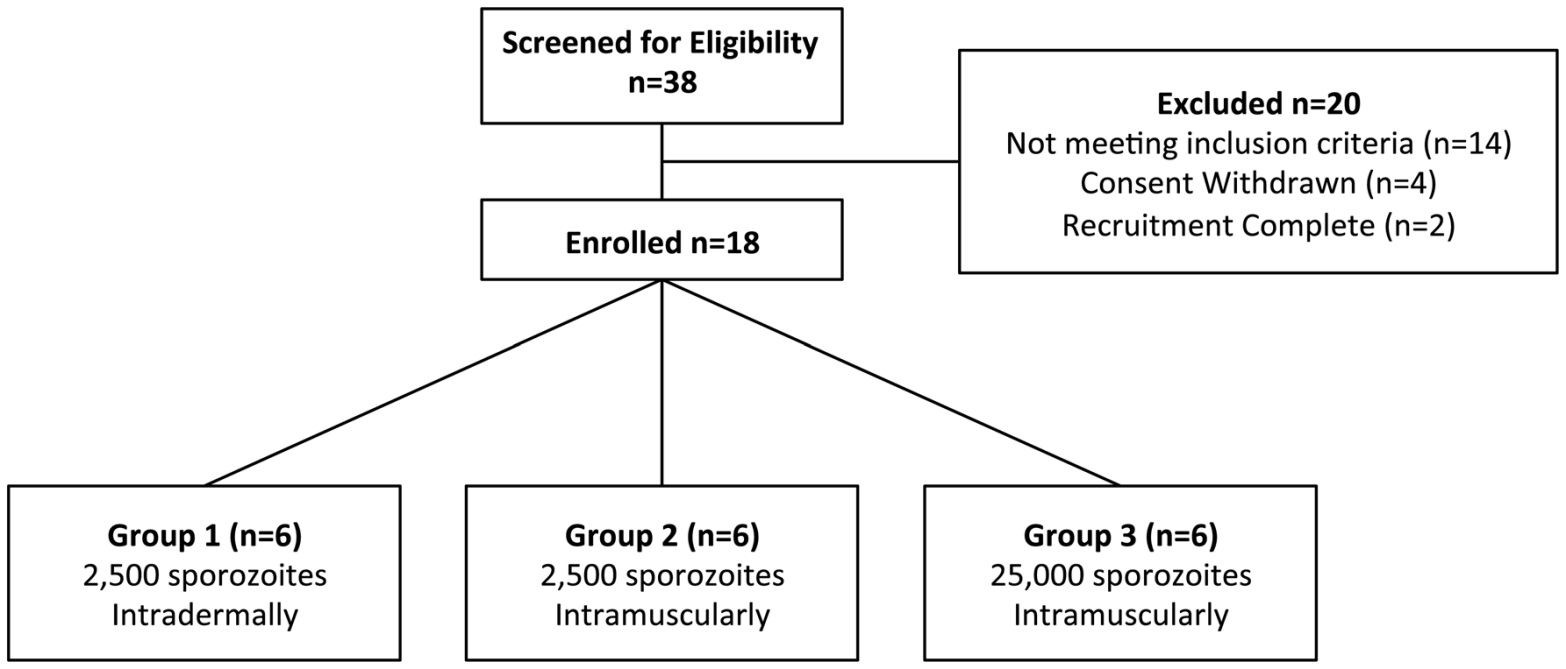
Flow chart of study design and volunteer recruitment. 20 participants were excluded following screening for the following reasons: significant psychiatric history (six individuals), consent withdrawn (four individuals), recruitment complete (2 individuals), lack of response from General Practitioner to medical screening letter (2 individuals), unexplained systolic murmur, elevated alanine transaminase, unexplained tachycardia and significant prior malaria exposure. In each group, the total dose of sporozoites was split between two injection sites and administered as two 50 µL injections, one in each deltoid.

The study was an open label, non-randomised pilot study with blinded laboratory outcome assessment (Figure 1). The study was conducted at the Centre for Clinical Vaccinology and Tropical Medicine, University of Oxford, UK. Healthy, malaria-naïve males and non-pregnant females aged 18–45 years were invited to participate in the study. All participants gave written informed consent, and the study was conducted according to the principles of the Declaration of Helsinki and in accordance with Good Clinical Practice (GCP) (see Materials & Methods S1 for the full list of inclusion and exclusion criteria). Allocation to study groups occurred at screening according to volunteer preference.

### Ethical & Regulatory Approval

Ethical approval was granted by the UK National Research Ethics Committee South Central Oxford A (Ref: 11/SC/0351). UK regulatory approval of the trial was not necessary as PfSPZ Challenge is considered a non-investigational product in the UK. The study was reviewed and allowed to proceed by the US Food and Drug Administration under IND 14267. The trial was registered with ClinicalTrials.gov (NCT01465048). The Local Safety Committee provided safety oversight and GCP compliance was independently monitored by the University of Oxford’s Department for Clinical Trials and Research Governance.

### Procedures

Aseptic cryopreserved *P. falciparum* sporozoites were manufactured according to Good Manufacturing Practice (GMP) standards by the biotechnology company Sanaria (USA) (see Materials & Methods S1). The manufacturing process was identical to that previously described for PfSPZ Vaccine [15], [28] with the exception that sporozoites in PfSPZ Challenge were not irradiated. Vials of PfSPZ Challenge were stored and transported to site in liquid nitrogen vapour phase. The same manufacturing lot of sporozoites was used for all participants. PfSPZ Challenge was thawed, mixed with diluent (phosphate buffered saline and human serum albumin) and prepared for injection at the study site under aseptic conditions by staff from Sanaria. The maximum interval allowed between thawing for PfSPZ Challenge and administration to participants was 30 minutes.

The 6 participants in Group 1 and 2 participants from group 2 were enrolled first on the same day. In the absence of safety concerns in these participants, 48 hours later the remaining 4 participants in Group 2 and 2 participants from Group 3 were enrolled. In the absence of safety concerns in these participants, 48 hours later the remaining participants in Group 3 were enrolled.

For each group the total dose of sporozoites was divided into two equal doses and administered as 50 µL injections, one in each deltoid (IM) or in the skin above the deltoid (ID). Details of dosing, clinical follow-up and safety monitoring are given in Materials & Methods S1 (Tables S1 & S2).

### Safety

Volunteers were reviewed in clinic the day after CHMI. All volunteers were called daily on days 2–5 post CHMI (C+2–5) to enquire as to adverse events and use of medications. As in previous mosquito bite CHMI studies at our centre, volunteers were reviewed in clinic on dC+6 in the evening and then twice a day, morning and evening between dC+7 and dC+14. Undiagnosed volunteers were reviewed once a day in the morning between dC+15 and C+21. At each visit, blood was sampled for microscopy and qPCR, physical observations performed and AEs solicited. AE were graded according to the criteria in Tables S1 & S2. On diagnosis, volunteers were treated with a 3 day curative course of oral Malarone (atovaquone/proguanil 250 mg/100 mg) where each dose was directly observed in clinic. Volunteers were reviewed 24 and 48 hours post diagnosis where blood was sampled for microscopy. Provided these two blood-films after treatment were negative for parasites volunteers were not reviewed again in clinic until dC+35. If one of these blood films was positive, volunteers continued to be reviewed in clinic at 24-hour intervals until two consecutive blood films were negative. Volunteers were then reviewed at dC+35 and dC+90 where safety assessments were performed. Full blood count with differential, platelet count and serum biochemistry (including electrolytes, urea, creatinine, bilirubin, alanine aminotransferase, alkaline phosphatase and albumin) were measured at screening, the day before CHMI, at visit dC+9, within 24 hours of diagnosis, and at visits on dC+35 and dC+90. Blood was drawn for immunology at visits on dC+7, d+C11 and C21 if persistently undiagnosed with malaria. If diagnosed with malaria, 70 mL of blood was drawn within 24 hours of diagnosis (but not if diagnosed on dC+7, dC+7.5, d+C11 or d+C11.5) and then no further blood drawn for explorative immunology until C35. Throughout the paper, study day refers to the nominal time point for a group and not the actual day of sampling.

### Malaria Diagnosis

Successful malaria infection was defined as positive thick film microscopy with at least one morphologically normal malaria trophozoite seen by one or more experienced microscopists in the presence of symptoms consistent with malaria in 200 high power fields (Table S3). Real time qPCR for *P. falciparum* was performed simultaneously, although clinical investigators were blinded to the results. If a volunteer described symptoms or displayed signs likely to represent malaria infection in the opinion of clinical investigators (such as fever, rigors or severe symptomatology), despite having a negative thick film and in the absence of an alternative cause, clinical investigators were un-blinded to the qPCR result. If this was positive the volunteer was treated for malaria. In the case of participants with positive thick film microscopy, but no symptoms consistent with *P. falciparum* malaria infection, clinical investigators were un-blinded to the qPCR results and the volunteer only treated if any preceding samples had >500 parasites per mL. This was to reduce the likelihood of false-positive diagnosis by microscopy. Time to diagnosis was measured in hours and converted to days for analysis.

### Parasite Growth Modelling

Quantitative real-time PCR (qPCR) was conducted as previously described. [29] Briefly, DNA was extracted from 0.5 mL blood, filtered to reduce WBC content, using Qiagen Blood Mini Kit. 10% of each extraction was run in triplicate qPCR – equivalent to 150 uL blood directly assessed. Parasites per mL equivalent mean values were generated by a standard Taqman absolute quantitation, against a defined plasmid standard curve with an ABi StepOne Plus machine and v2.1 software using default Universal qPCR and QC settings, apart from the use of 45 cycles and 25 uL reaction volume. Based upon results obtained using a dilution series of microscopically-counted cultured parasites this method has a lower limit of quantification (LLQ, defined as %CV<20%) of around 20 parasites/mL blood (p/mL). [30] The number of infected erythrocytes in the first generation after parasite release from the liver (liver-to-blood inoculum, LBI) and the parasite multiplication rate in the blood (PMR) were estimated (full modelling details are provided in Materials & Methods S1). Time in hours between challenge and collection of each blood-sample was calculated using data specific to each volunteer for each of these events and then converted to days. Data from non-immunised infectivity control subjects from three previous mosquito-bite CHMI trials were used as comparators (*Ewer et al. unpublished*). [29].

### *Ex-vivo* Interferon-γ (IFN-γ) ELIspot

Blood peripheral blood mononuclear cell (PBMC) ELISpot assays were performed as previously described. [31] Briefly PBMCs were isolated after centrifugation over Lymphoprep gradients followed by culturing 250,000 PBMCs per well with the relevant peptide at a final concentration of 1 µg/mL (Neo Group Inc., USA, Mimotopes, Australia and Thermo Fischer Scientific, USA) on anti-IFN-γ coated plates. After 18–20 hours, plates were developed as previously described. [31] IFN-γ spot forming units (SFU) were enumerated using an ELISpot counter (Autoimmun Diagnostika, Germany) with the results presented as SFU per million PBMCs after the background (response to media only) and responses at day before CHMI (C-1) were subtracted. Antigens to assess IFN-γ production were chosen based on reported findings in the literature; they were either a) pre-erythrocytic liver and blood stage antigens previously assessed for vaccine induced T cell immunogenicity (MSP, [31], [32] AMA, [32], [33] TRAP, [34] Pfs16, STRAP, EXP1, LSA1 [35]), b) targets of immune responses in naturally exposed individuals or volunteers vaccinated with irradiated sporozoites (CelTOS, [26] Exp1, [36], [37], LSA1 [24], [38] and LSA3 [39], STARP [40], Pfs16 [41]), c) proteins known to have protective homologs based on murine or non-human primate sub-unit vaccination studies (CelTOS, [42] Exp1, [43] LSA3, [36], [44] PfUIS3, [45] PFI0580c [45]), or d) proteins recently identified as highly up-regulated during the liver-stage (LSAP1, [46] LSAP2 [46] and PFE1590w). [47].

### Statistical Analysis

The study was designed to assess proof of concept rather than to look for statistically significant associations and the sample size was accordingly kept to the minimum. As is appropriate in an underpowered study, statistical analyses are primarily descriptive in nature and significance tests used sparingly and interpreted with caution. Data were analyzed using GraphPad Prism version 5.03 for Windows (GraphPad Software Inc., USA). Differences between groups in time to malaria diagnosis were compared using a log-rank test and described using the Kaplan Meier method. qPCR results were compared using non-parametric tests.

## Results

### Study Participants and Intervention

Recruitment took place between September and November 2011. Eighteen healthy malaria-naïve adult participants (8 female and 10 male) underwent CHMI in November 2011 (Figure 1). The mean age of participants was 24.2 years (range 18–37) (Table S4).

All participants received injection of PfSPZ Challenge as scheduled with the exception of one volunteer in Group 1 who received approximately 10% less than the scheduled dose due to some of the inoculum leaking from the administration site post injection. All participants completed the study as scheduled. The mean time between thawing of PfSPZ Challenge and administration was 15.9 minutes (range 12–22) (Table S5).

### Infectivity of PfSPZ Challenge

**Figure 2:**
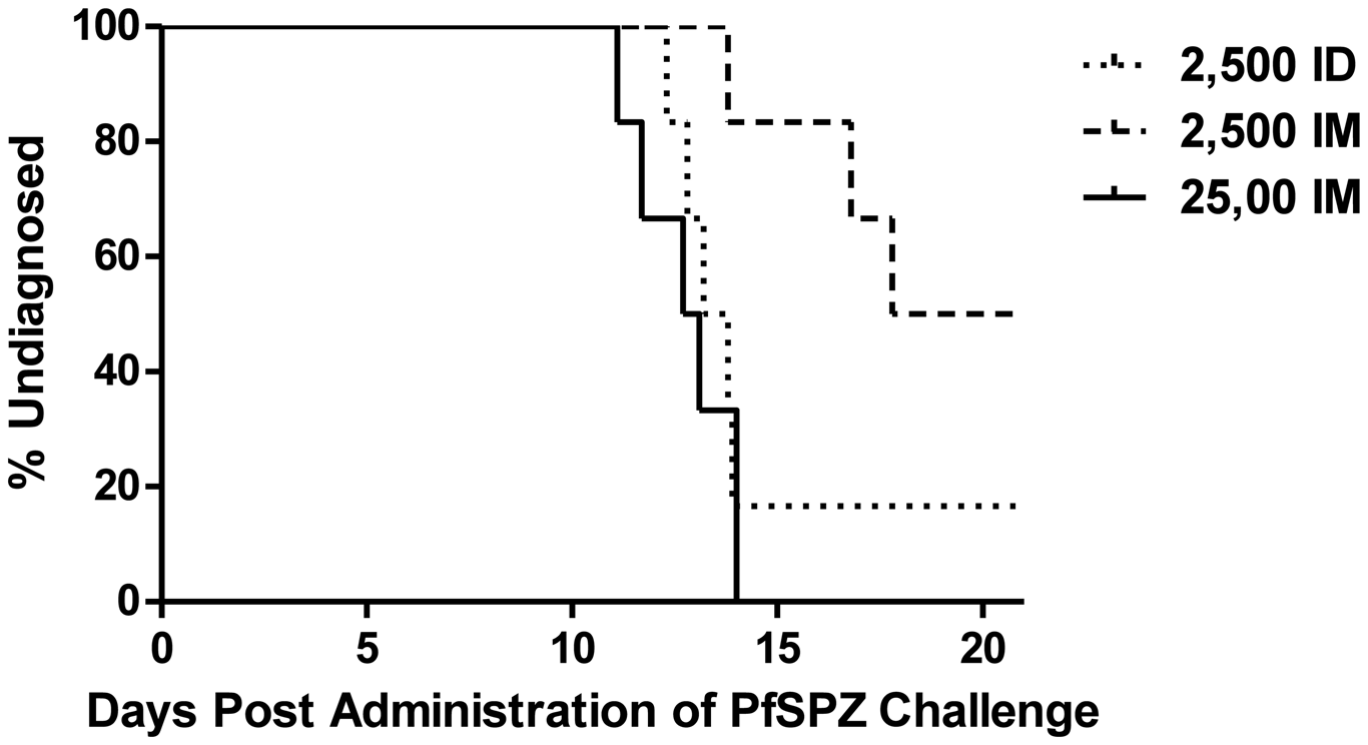
PfSPZ Challenge Infectivity Data. Kaplan-Meier analysis of time to patent parasitemia in days between injection and diagnosis (*p = 0.024* logrank test). 2,500 ID = 2,500 sporozoites administered intradermally (ID). 2,500 IM = 2,500 sporozoites administered intramuscularly. 25,000 = 25,000 sporozoites administered intramuscularly. Median pre-patent period = 13.19 days for 2,500 sporozoites ID, 17.8 days for 2,500 sporozoites IM, 12.72 days for 25,000 sporozoites IM.

Five out of six participants receiving 2,500 sporozoites ID in Group 1, 3/6 participants receiving 2,500 sporozoites IM in Group 2 and 6/6 participants receiving 25,000 sporozoites IM in Group 3 were successfully infected with malaria (Figure 2 and Table S6). Of note, the participant in Group 1 who received a lower dose of PfSPZ Challenge than planned was not infected with malaria. The median time to diagnosis was 13.2, 17.8 and 12.7 days for 2,500 sporozoites ID, 2,500 sporozoites IM and 25,000 sporozoites IM respectively (Kaplan Meier analysis; *p = 0.024* log rank test).

### Modelling of Parasitemia Measured by qPCR

**Figure 3:**
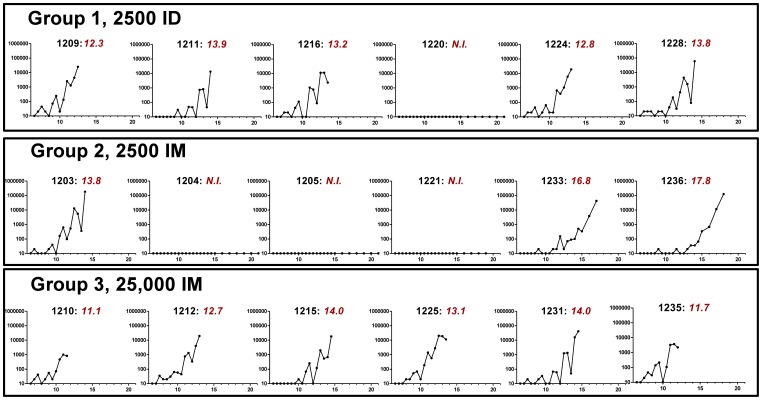
qPCR-measured parasite density for each individual subject grouped by dosing regimen. Y axis = qPCR. X axis = days post injection of PfSPZ Challenge. Black subtitles indicate subject identification numbers; red subtitles indicate time to diagnosis by thick film microscopy in days. NI = not infected.

**Figure 4:**
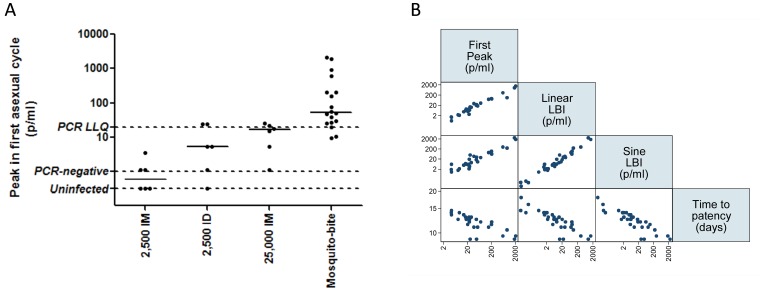
Estimation of Burden of Liver Infection and Liver-to-Blood Inoculum. Data from participants successfully infected with malaria in groups 1, 2 and 3 compared to historical data from mosquito bite CHMI trials undertaken at our centre. SpZ = sporozoites. LLQ = lower limit of quantification by qPCR. ID = intradermal administration. IM = intramuscular administration. Mosquito bite = malaria naïve participants infected with *P.falciparum* by mosquito bite as infectivity control participants in vaccine efficacy studies undertaken recently at our centre (*Ewer et al. submitted)*. [29] (A) Peak qPCR-measured parasitaemia in first asexual cycle for each regimen. (B) Matrix scatterplot illustrating close correlation of different LBI measures with each other and with time to microscopic patency.

Figure 3 plots the qPCR results for each individual in the trial. No positive results were obtained from any of the 82 blood samples (246 individual replicate qPCR reactions; Table S7) from the four individuals who were not diagnosed with malaria. All participants were qPCR-negative at samples taken 6.5 days post infection (dC+6.5) and so modelling commenced at dC+7. LBI calculated using a number of methods (Figure 4) [48], [49] were comparable between 2,500 sporozoites ID and 25,000 sporozoites IM. In agreement with pre-patent periods LBI results differed significantly across groups with 25,000 sporozoites IM having the highest LBI, followed by 2,500 sporozoites ID and 2,500 sporozoites IM (*P = 0.03* by Kruskal Wallis test). Of note, the PfSPZ dosing regimens led to lower LBI compared to mosquito bite CHMI trials at our centre (*P = 0.0001* Wilcoxon Rank Sum test for n = 18 historical mosquito bite controls compared to n = 18 PfSPZ participants combined; Figure 4A).

**Figure 5:**
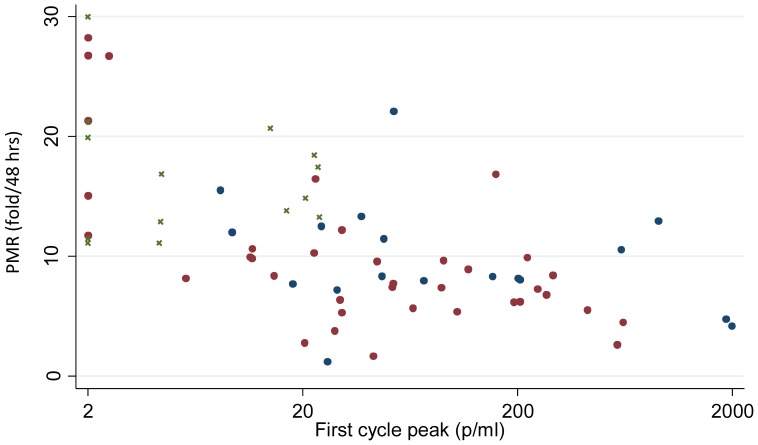
Parasite Multiplication Rate following PfSPZ Challenge is comparable to mosquito-bite subjects with similar LBIs. Figure shows relationship Between PMR and LBI for participants successfully infected with malaria in groups 1–3 (green crosses), compared to historical data from mosquito bite CHMI trials undertaken at our centre involving malaria naïve unvaccinated subjects (blue dots), and malaria naïve ChAd63-MVA ME-TRAP vaccinated subjects (red dots). These data were obtained using the linear model; results were similar with the sine model (data not shown).

On initial examination, estimated PMRs appeared higher following PfSPZ Challenge than for control subjects infected by mosquito bite (mean 16±5 fold parasite increase per 48 hours for PfSPZ Challenge; mean 10±4.7 fold parasite increase per 48 hours for mosquito-bite; Figure 5 & Figure S2). We have however recently found a negative relationship between LBI and PMR in mosquito-bite CHMI subjects, the reason for which remains unclear. [30] The distribution of estimated PMRs observed in participants infected using PfSPZ Challenge appears similar to that observed among subjects with low LBIs from previous mosquito-bite CHMI trials (principally subjects who had been immunised with the liver-stage antigen TRAP, which is unlikely to affect PMR; Figure 5). It thus seems likely that asexual parasite growth after PfSPZ Challenge administration follows similar kinetics to that after mosquito-bite challenge delivery of a similar LBI.

### PfSPZ Challenge Reactogenicity

All AEs following injection of PfSPZ Challenge were mild and short lived (Figure S1 and Table S8). No serious AEs occurred. One participant who received 25,000 sporozoites IM described gum bleeding on teeth-brushing starting the evening following injection of PfSPZ Challenge. Full blood count and clotting assays were normal and there was no visible pathology on examination. This AE resolved within 2 days and did not recur. No laboratory AEs related to PfSPZ Challenge injection were noted.

### PfSPZ Induced Clinical *Plasmodium falciparum* Infection (Figure S1)

One of the 14 participants (7.14%) diagnosed with *P. falciparum* malaria experienced no symptoms or signs of malaria infection. The total duration of symptoms in participants with symptomatic malaria infection ranged from 1–12 days (mean 6.0+/−3.12 days), similar to that seen at a recent mosquito bite CHMI trial at our centre, [29] and there appeared no difference in duration of symptoms of malaria between the groups. Two volunteers diagnosed with malaria (14.3%) had a fever prior to diagnosis (38.2°C & 38.3°C) and one volunteer developed a fever post-treatment (37.6°C). Nine out of 14 participants diagnosed with *P. falciparum* (64.29%) experienced at least one AE post challenge that was severe in intensity. There were no serious AEs. No participants were admitted for in-patient management of malaria infection. Safety bloods taken at dC+9, dC+35, dC+90 and within 24 hours of diagnosis demonstrated transient laboratory abnormalities at frequencies and severities expected following *P. falciparum* infection. [29], [50].

### T Cell Immunogenicity Assessed by *ex-vivo* IFN-γ ELIspot

**Figure 6:**
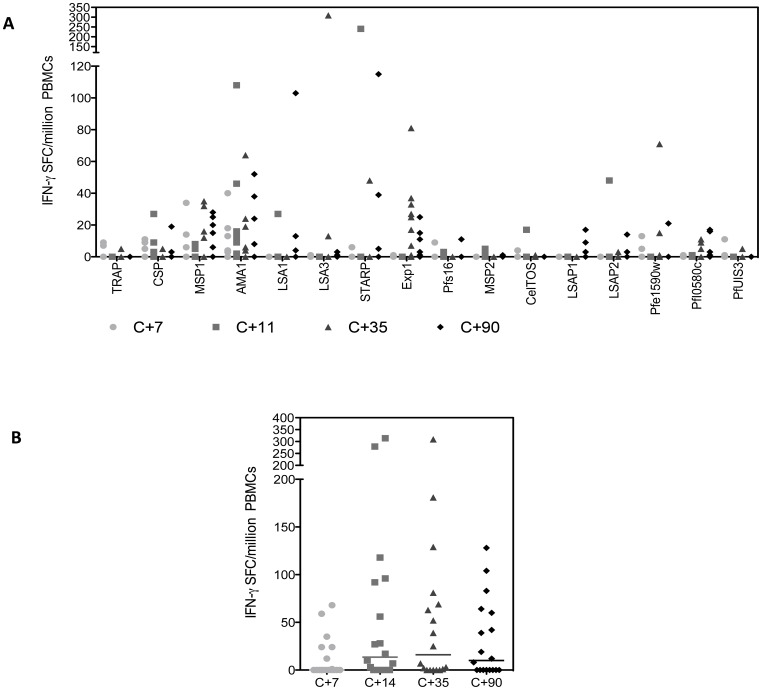
IFN-γ ELISPOT responses to individual malaria antigens post CHMI. Median *ex-vivo* IFN-γ ELISPOT responses from the day before CHMI (C-1) and days 7, 11, 14, 35 and 90 post CHMI with cells stimulated against peptide pools to numerous malaria antigens. Each volunteer is represented as a single point. Lines represent the median response at each time point. (A) Individual responses to numerous malaria antigens over time (B) Sum responses to all peptides over time.

A modest response (>50 SFC per million PBMCs) was seen to each antigen in at least one volunteer at one time point (Figure 6), although it is difficult to be sure that some of these apparently positive responses were not chance findings. The same pattern of immunogenicity was observed in all three groups (data not shown) and therefore the responses to each antigen were pooled together. Responses tended to increase over time up to dC+35 followed by an overall decrease by dC+90. The greatest response rate to a single antigen was 43% of all participants responding to EXP1 at dC+35.

## Discussion

We have shown that PfSPZ Challenge is a potent and safe product capable of inducing *P. falciparum* infection in malaria naïve individuals. In those participants who developed symptomatic *P. falciparum* infection, the severity and duration of symptoms were reassuringly similar to those seen following CHMI administered by mosquito bite at our centre, [29] and no concerning AEs were noted following injection of PfSPZ Challenge.

Screening of multiple pre-erythrocytic stage antigens using ELIspot analysis failed to identify any clear immunodominant antigens. T-cell immunogenicity at dC+35 was of a similar magnitude to that previously reported from individuals from malaria endemic areas and in some cases to individuals who received multiple doses of irradiated sporozoites [15], [21], [27], although the proportion of individuals responding to some antigens like PfCelTOS (Ag2) was significantly lower than after immunization by the bite of irradiated PfSPZ-infected mosquitoes. [26] The decrease in T-cell immunogenicity by dC+90 supports the notion that repeated low-level antigen exposure would be required to induce and maintain IFN-γ responses of a significant protective magnitude.

This is the second study to assess the infectivity of 2,500 sporozoites of PfSPZ Challenge administered ID. Our result for this dosing schedule (5/6 participants successfully infected, pre-patent period 13.2 days) is near identical to that seen by *Roestenberg et al.* (5/6 participants successfully infected, pre-patent period 13.0 days). [12] Given that different batches of PfSPZ Challenge, manufactured two years apart, were used for each of these trials, this result demonstrates the reproducibility of PfSPZ Challenge as a challenge agent.

For the first time we have identified an administration regimen for PfSPZ Challenge (25,000 sporozoites IM) that successfully infects 100% of participants. However, given there was no difference in pre-patent period or LBI between 25,000 sporozoites IM and 2,500 sporozoites ID it is likely that the increased proportion of individuals successfully infected with 25,000 sporozoites IM is consistent with chance.

In contrast to murine data, administration of 2,500 sporozoites ID was associated with higher infectivity (in terms of proportion of participants infected, duration of pre-patent period and LBI) than 2,500 sporozoites administered IM, suggesting ID administration is a more effective route for delivering sporozoites to the liver in humans. The reason for the discrepancy between murine and human models is not clear; however, this finding might be intuitive given that ID injection by needle and syringe most closely resembles injection via a mosquito’s proboscis. Alternatively, other factors such as administration volume could impact on infectivity.

In our study, a dose response was seen when PfSPZ Challenge was administered IM, with 25,000 sporozoites appearing more infectious than 2,500. This is in contrast to the study by *Roestenberg et al.* where increasing the dose of sporozoites ID failed to translate into an increase in the proportion of participants successfully infected. [12] The reason for this difference is unclear but supports the evaluation of higher doses of PfSPZ Challenge IM, which may, in contrast to ID administration, lead to identification of a dose that reliably infects 100% of participants.

The pre-patent periods of infected participants in our trial were longer than those seen in participants undergoing CHMI by mosquito bite at our centre. This and our parasite modelling data support the conclusion that PfSPZ Challenge administered by needle and syringe in the dosing regimens we have evaluated is not as effective at delivering sporozoites to the liver as five mosquito bites. Future dose and route finding studies should seek to identify dosing regimens that not only reliably infect 100% of participants but that produce pre-patent periods similar to those in CHMI studies administered by mosquito bite. This work will include evaluating the effect of varying the number of administration sites and volume of inoculum, both of which affect infectivity of cryopreserved sporozoites pre-clinically. [13] Our data should not only guide future trials to optimise PfSPZ Challenge as a CHMI method but also help inform dosing decisions regarding promising whole sporozoite vaccines [15], [51], [52].

## Supporting Information

Figure S1 **Analysis of Clinical Data.** **(A)** AEs deemed definitely, probably or possibly related to PfSPZ Challenge injection (excluding symptoms related to result *P. falciparum* infection). Data are combined for all AEs for all volunteers receiving the same dose of PfSPZ. There were no serious AEs. **(B)** Comparison of duration of symptoms and signs associated related to malaria in individuals who were diagnosed with malaria (n = 14) (P = 0.073). Duration of symptoms in group 1: mean 5.8 days, median 6.0 days. Duration of symptoms in group 2: mean 9.0 days, median 9.0 days. Duration of symptoms in group 3: mean 3.7 days, median 4.0 days. Median values for each group are indicated on the figure. **(D)** Comparison of maximum severity of any AE deemed possibly, probably or definitely related to malaria infection in individuals diagnosed with malaria (excluding laboratory AEs) (*n* = 14). **(E)** Laboratory AEs post CHMI deemed possibly, probably or definitely related to *P. falciparum* infection. ALT = Alanine transaminase. For ‘any laboratory abnormality’ only the highest intensity laboratory AE per subject is counted.
(TIF)

Figure S2 **Comparing qPCR data with Data from Mosquito Bite CHMI at same centre.** Red dots: qPCR-measured parasite density for each individual subject in current trial and unimmunised control subjects from three previous mosquito-bite CHMI trials. Blue line: linear model-fitted parasite growth kinetic. Green horizontal line: linear-model estimated LBI. Red vertical line indicates time at which liver release is considered to be complete and hence LBI is estimated (day 7.5). Black subtitles indicate challenge regime, subject ID numbers, and trial (VAC049 = current trial; MAL034A, MAL034B and VAC039 = previous mosquito bite challenges).
(TIF)

Table S1 **Criteria for Grading Severity of Local AEs Related to PfSPZ Challenge Injection.**
(DOCX)

Table S2 **Functional Criteria for Grading Severity of Systemic AEs.**
(DOCX)

Table S3 **Criteria for Malaria Diagnosis.**
(DOCX)

Table S4 **Demographics of Enrolled Volunteers.**
(DOCX)

Table S5 **Time between Thawing of PfSPZ Challenge and Administration (minutes).**
(DOCX)

Table S6 **End Points for Treatment of Subjects. BF = blood film.**
(DOCX)

Table S7 **Raw qPCR data (parasites/mL).** Top row represents day of follow-up visit post administration of PfSPZ Challenge. N = PCR negative (i.e. <20 parasites/mL) highlighted in grey. Squares coloured red represent point of diagnosis
(DOCX)

Checklist S1 **CONSORT Checklist.**
(DOC)

Materials & Methods S1 (DOC)

Protocol S1 Study protocol.
(PDF)
