# Supplementary material for: Optimising Controlled Human Malaria Infection Studies Using Cryopreserved P. falciparum Parasites Administered by Needle and Syringe
Source: PLoS One. 2013 Jun 18;8(6):e65960. doi: 10.1371/journal.pone.0065960 (PMC3688861; doi:10.1371/journal.pone.0065960)
Supplement: Table S1 — Criteria for Grading Severity of Local AEs Related to PfSPZ Challenge Injection. (DOCX) [file pone.0065960.s003.docx]

**Table S1: Criteria for Grading Severity of Local AEs Related to PfSPZ Challenge Injection**

| **Adverse Event** | **Grade** | **Intensity** |
| --- | --- | --- |
| Pain at injection site | 1 | Pain that is easily tolerated |
|  | 2 | Pain that interferes with daily activity |
|  | 3 | Pain that prevents daily activity |
| Erythema at injection site* | 1 | >3 - ≤50 mm |
|  | 2 | >50 - ≤100 mm |
|  | 3 | >100 mm |
| Swelling at injection site | 1 | >0 - ≤20 mm |
|  | 2 | >20 - ≤50 mm |
|  | 3 | >50 mm |
