# Supplementary material for: Optimising Controlled Human Malaria Infection Studies Using Cryopreserved P. falciparum Parasites Administered by Needle and Syringe
Source: PLoS One. 2013 Jun 18;8(6):e65960. doi: 10.1371/journal.pone.0065960 (PMC3688861; doi:10.1371/journal.pone.0065960)
Supplement: Table S2 — Functional Criteria for Grading Severity of Systemic AEs. (DOCX) [file pone.0065960.s004.docx]

**Table S2: Functional Criteria for Grading Severity of Systemic AEs**

| **GRADE 0** | None |
| --- | --- |
| **GRADE 1** | Mild: Transient or mild discomfort (< 48 hours); no medical intervention/therapy required |
| **GRADE 2** | Moderate: Mild to moderate limitation in activity - some assistance may be needed; no or minimal medical intervention/therapy required |
| **GRADE 3** | Severe: Marked limitation in activity, some assistance usually required; medical intervention/therapy required, hospitalisation possible |
