# Supplementary material for: Optimising Controlled Human Malaria Infection Studies Using Cryopreserved P. falciparum Parasites Administered by Needle and Syringe
Source: PLoS One. 2013 Jun 18;8(6):e65960. doi: 10.1371/journal.pone.0065960 (PMC3688861; doi:10.1371/journal.pone.0065960)
Supplement: Table S3 — Criteria for Malaria Diagnosis. (DOCX) [file pone.0065960.s005.docx]

**Table S3: Criteria for Malaria Diagnosis**

|  | **THICK FILM MICROSCOPY** | |
| --- | --- | --- |
| **MALARIAL SYMPTOMS** | **Positive** | **Negative** |
| **Symptomatic** | Successfully Infected | Successfully infected if any available PCR result is > 500 parasites/ml |
| **Asymptomatic** | Successfully infected if any available PCR result is > 500 parasites/ml  (Otherwise delay treatment) | Not infected |
