# Supplementary material for: Optimising Controlled Human Malaria Infection Studies Using Cryopreserved P. falciparum Parasites Administered by Needle and Syringe
Source: PLoS One. 2013 Jun 18;8(6):e65960. doi: 10.1371/journal.pone.0065960 (PMC3688861; doi:10.1371/journal.pone.0065960)
Supplement: Table S4 — Demographics of Enrolled Volunteers. (DOCX) [file pone.0065960.s006.docx]

**Table S4. Demographics of Enrolled Volunteers**

|  | **2,500 ID** | **2.500 IM** | **25,000 IM** |
| --- | --- | --- | --- |
|  | **n = 6** | **n = 6** | **n = 6** |
| ***Sex*** | | | |
| Male | 3 | 3 | 4 |
| Female | 3 | 3 | 2 |
| ***Age at screening (years)*** | | | |
| Mean ± SD | 23.8 +/- 5.0 | 26.2 +/- 7.6 | 22.5 +/- 3.0 |
| Median | 22.00 | 23.50 | 21.00 |
| Min, Max | 20, 33 | 18, 37 | 20, 28 |
