# Supplementary material for: Optimising Controlled Human Malaria Infection Studies Using Cryopreserved P. falciparum Parasites Administered by Needle and Syringe
Source: PLoS One. 2013 Jun 18;8(6):e65960. doi: 10.1371/journal.pone.0065960 (PMC3688861; doi:10.1371/journal.pone.0065960)
Supplement: Table S5 — Time between Thawing of PfSPZ Challenge and Administration (minutes). (DOCX) [file pone.0065960.s007.docx]

**Table S5. Time between Thawing of PfSPZ Challenge and Administration (minutes)**

|  | **2,500 ID** | **2,500 IM** | **25,000 IM** |
| --- | --- | --- | --- |
|  | **n = 6** | **n = 6** | **n = 6** |
| Mean ± SD | 15.50 +/- 2.429 | 17.17 +/- 3.061 | 15.00 +/- 1.414 |
| Median | 16.50 | 17.50 | 15.00 |
| Min, Max | 12, 18 | 13, 22 | 13, 17 |
