# Supplementary material for: Optimising Controlled Human Malaria Infection Studies Using Cryopreserved P. falciparum Parasites Administered by Needle and Syringe
Source: PLoS One. 2013 Jun 18;8(6):e65960. doi: 10.1371/journal.pone.0065960 (PMC3688861; doi:10.1371/journal.pone.0065960)
Supplement: Table S6 — End Points for Treatment of Subjects. BF = blood film. (DOCX) [file pone.0065960.s008.docx]

**Table S6: End Points for Treatment of Subjects.** BF = blood film.

|  | **2,500 ID**  **n=6** | **2,500 IM**  **n=6** | **25,000 IM**  **n=6** |
| --- | --- | --- | --- |
| Symptomatic with positive BF | 1 (17%) | 2 (33%) | 2 (33%) |
| Symptomatic with negative BF | 0 | 0 | 0 |
| Asymptomatic with positive BF & PCR > 500 parasites/ml | 4 (66%) | 1 (17%) | 4 (66%) |
| Reached Day 21 undiagnosed (asymptomatic, BF & PCR negative) | 1 (17%) | 3 (50%) | 0 |
| Withdrew Consent or Excluded From Study Prior to Primary Endpoint | 0 | 0 | 0 |
