# Supplementary material for: Optimising Controlled Human Malaria Infection Studies Using Cryopreserved P. falciparum Parasites Administered by Needle and Syringe
Source: PLoS One. 2013 Jun 18;8(6):e65960. doi: 10.1371/journal.pone.0065960 (PMC3688861; doi:10.1371/journal.pone.0065960)
Supplement: Table S7 — Raw qPCR data (parasites/mL). Top row represents day of follow-up visit post administration of PfSPZ Challenge. N = PCR negative (i.e. <20 parasites/mL) highlighted in grey. Squares coloured red represent point of diagnosis (DOCX) [file pone.0065960.s009.docx]

**Table S7**. **Raw qPCR data (parasites/mL)** Top row represents day of follow-up visit post administration of PfSPZ Challenge. N = PCR negative (i.e. <20 parasites/mL) highlighted in grey. Squares coloured red represent point of diagnosis.

|  | Volunteer no. | C6.5 | C7 | C7.5 | C8 | C8.5 | C9 | C9.5 | C10 | C10.5 | C11 | C11.5 | C12 | C12.5 | C13 | C13.5 | C14 | C14.5 | C15 | C16 | C17 | C18 | C19 | C20 | C21 |
| --- | --- | --- | --- | --- | --- | --- | --- | --- | --- | --- | --- | --- | --- | --- | --- | --- | --- | --- | --- | --- | --- | --- | --- | --- | --- |
| **2,500 ID** | 1209 | neg | neg | 34 | neg | neg | 62 | 232 | neg | 121 | 2491 | 1269 | 4348 | 25033 |  |  |  |  |  |  |  |  |  |  |  |
|  | 1211 | neg | neg | neg | neg | neg | neg | 21 | neg | neg | 39 | 36 | neg | 720 | 807 | 36 | 13523 |  |  |  |  |  |  |  |  |
|  | 1216 | neg | neg | neg | neg | neg | 32 | 103 | neg | neg | 1047 | 766 | 78 | 11209 | 11234 | 2327 |  |  |  |  |  |  |  |  |  |
|  | 1220 | neg | neg | neg | neg | neg | neg | neg | neg | neg | neg | neg | neg | neg | neg | neg | neg | neg | neg | neg | neg | neg | neg | neg | neg |
|  | 1224 | neg | neg | neg | 35 | neg | neg | 54 | neg | neg | 660 | 386 | 1044 | 6031 | 19075 |  |  |  |  |  |  |  |  |  |  |
|  | 1228 | neg | neg | neg | neg | neg | neg | neg | neg | 28 | 178 | 21 | 413 | 4316 | 1565 | 68 | 59262 |  |  |  |  |  |  |  |  |
|  |  |  |  |  |  |  |  |  |  |  |  |  |  |  |  |  |  |  |  |  |  |  |  |  |  |
| **2,500 IM** | 1203 | neg | neg | neg | neg | neg | neg | 30 | neg | 153 | 617 | 89 | 526 | 12995 | 5538 | 352 | 185818 |  |  |  |  |  |  |  |  |
|  | 1204 | neg | neg | neg | neg | neg | neg | neg | neg | neg | neg | neg | neg | neg | neg | neg | neg | neg | neg | neg | neg | neg | neg | neg | neg |
|  | 1205 | neg | neg | neg | neg | neg | neg | neg | neg | neg | neg | neg | neg | neg | neg | neg | neg | neg | neg | neg | neg | neg | neg | neg | neg |
|  | 1221 | neg | neg | neg | neg | neg | neg | neg | neg | neg | neg | neg | neg | neg | neg | neg | neg | neg | neg | neg | neg | neg | neg | neg | neg |
|  | 1233 | neg | neg | neg | neg | neg | neg | neg | neg | neg | neg | neg | 143 | neg | 60 | 80 | 92 | 497 | 328 | 3823 | 42902 |  |  |  |  |
|  | 1236 | neg | neg | neg | neg | neg | neg | neg | neg | neg | neg | neg | neg | neg | neg | 26 | 26 | 56 | 329 | 666 | 11586 | 126259 |  |  |  |
|  |  |  |  |  |  |  |  |  |  |  |  |  |  |  |  |  |  |  |  |  |  |  |  |  |  |
| **25,000 IM** | 1210 | neg | neg | 31 | neg | neg | 45 | neg | 62 | 456 | 1000 | 820 |  |  |  |  |  |  |  |  |  |  |  |  |  |
|  | 1212 | neg | neg | 25 | neg | neg | 21 | 54 | 48 | 35 | 745 | 1302 | 326 | 4026 | 19715 |  |  |  |  |  |  |  |  |  |  |
|  | 1215 | neg | neg | neg | neg | neg | neg | neg | neg | neg | 58 | 246 | neg | 111 | 1974 | 528 | 666 | 18139 |  |  |  |  |  |  |  |
|  | 1225 | neg | neg | neg | neg | neg | 42 | 59 | neg | 178 | 1396 | 558 | 2798 | 20309 | 18951 | 11380 |  |  |  |  |  |  |  |  |  |
|  | 1231 | neg | neg | neg | neg | neg | neg | 24 | neg | neg | 58 | 51 | neg | 1227 | 1317 | 41 | 15954 | 41087 |  |  |  |  |  |  |  |
|  | 1235 | neg | neg | neg | 36 | 20 | 128 | 204 | neg | 98 | 3289 | 3695 | 2214 |  |  |  |  |  |  |  |  |  |  |  |  |
