# Supplementary material for: Optimising Controlled Human Malaria Infection Studies Using Cryopreserved P. falciparum Parasites Administered by Needle and Syringe
Source: PLoS One. 2013 Jun 18;8(6):e65960. doi: 10.1371/journal.pone.0065960 (PMC3688861; doi:10.1371/journal.pone.0065960)
Supplement: Materials & Methods S1 — (DOC) [file pone.0065960.s011.doc]

**SUPPLEMENTARY MATERIALS & METHODS**

**Inclusion Criteria**

Volunteer must satisfy all the following criteria to be eligible for the study:

- Healthy adults aged 18 to 45 years.
- Able and willing (in the Investigator’s opinion) to comply with all study requirements.
- Willing to allow the investigators to discuss the volunteer’s medical history with their General Practitioner.
- Women only: Must practice continuous effective contraception for the duration of the study.
- Agreement to refrain from blood donation during the course of the study and for at least 3 years after the end of their involvement in the study.
- Written informed consent to undergo CHMI.
- Reachable (24/7) by mobile phone during the whole study period.
- Willingness to take a curative anti-malaria regimen.
- For volunteers not living in Oxford: agreement to stay in a hotel room close to the trial centre during a part of the study (At least Day 6.5 post inoculation until 2 days after treatment commenced).
- Answer all questions on the informed consent quiz correctly.

**Exclusion Criteria**

Volunteers may not enter the study if any of the following apply:

- History of clinical *P. falciparum* malaria.
- Travel to a malaria endemic region during the study period or within the preceding six months with positive *P. falciparum* serology at screening.
- Use of systemic antibiotics with known antimalarial activity within 30 days of study enrolment (e.g. trimethoprim-sulfamethoxazole, doxycycline, tetracycline, clindamycin, erythromycin, fluoroquinolones and azithromycin)
- Receipt of an investigational product in the 30 days preceding enrolment, or planned receipt during the study period.
- Prior receipt of an investigational malaria vaccine.
- Any confirmed or suspected immunosuppressive or immunodeficient state, including HIV infection; asplenia; recurrent, severe infections and chronic (more than 14 days) immunosuppressant medication within the past 6 months (inhaled and topical steroids are allowed).
- Use of immunoglobulins or blood products within 3 months prior to enrolment.
- History of sickle cell anaemia, sickle cell trait, thalassemia or thalassemia trait.
- Pregnancy, lactation or intention to become pregnant during the study.
- A history of allergic disease or reactions likely to be exacerbated by malaria infection.
- Contraindications to the use of all three proposed anti-malarial medications; Malarone, Riamet and Chloroquine.
- History of cancer (except basal cell carcinoma of the skin and cervical carcinoma in situ).
- History of serious psychiatric condition that may affect participation in the study.
- Any other serious chronic illness requiring hospital specialist supervision.
- Suspected or known current alcohol abuse as defined by an alcohol intake of greater than 42 units every week.
- Suspected or known injecting drug abuse in the 5 years preceding enrolment.
- Seropositive for hepatitis B surface antigen (HBsAg).
- Seropositive for hepatitis C virus (antibodies to HCV).
- An estimated, ten year risk of fatal cardiovascular disease of ≥5%, as estimated by the Systematic Coronary Risk Evaluation (SCORE) system.
- Positive family history in 1st and 2nd degree relatives < 50 years old for cardiac disease.
- Volunteers unable to be closely followed for social, geographic or psychological reasons.
- Any clinically significant abnormal finding on biochemistry or haematology blood tests, urinalysis or clinical examination.
- Any other significant disease, disorder or finding which may significantly increase the risk to the volunteer because of participation in the study, affect the ability of the volunteer to participate in the study or impair interpretation of the study data.

**Exclusion Criterion on Day of Challenge**

- Acute disease, defined as moderate or severe illness with or without fever.

**Backup Volunteers**

In addition to the 18 volunteers to be enrolled in the study, back-up volunteers who were screened and were eligible to participate in the study were identified. These volunteers were available to be enrolled in the study at short notice should a planned volunteer withdraw consent or become in-eligible immediately prior to CHMI.

**PfSPZ Challenge Manufacture & Preparation for Administration**

Manufacture includes the production, under traditional environmental conditions, of eggs from a colony of *A. stephensi* mosquitoes housed in a controlled environmental chamber. Surface disinfection of the eggs is performed by exposure to chemical agents in a Class II biosafety cabinet (BSC). From this point forward, all materials and product are handled using aseptic methods to ensure that contaminating microorganisms are not introduced to and carried through the process. Surface-disinfected eggs are inoculated into sterile, vented flasks containing aseptic growth medium. The eggs hatch and develop into pupae, which are transferred to an adult mosquito container where the adult mosquitoes emerge. These adult mosquitoes, which have been raised under aseptic conditions, are fed *P. falciparum* gametocyte-infected blood in a BSC in a High-Security Insectary in Rockville, Maryland, USA. The *P. falciparum* gametocyte-infected blood has been produced from cultures of the *P. falciparum* strain NF54 derived from a Master Cell Bank of the well-characterized *P. falciparum* strain NF54. Infected adult mosquitoes are maintained under aseptic conditions until *P. falciparum* sporozoites migrate to the salivary glands. The salivary glands from the *P. falciparum* sporozoite infected mosquitoes are removed by hand dissection. Salivary glands are then triturated to release the *P. falciparum* sporozoites. The sporozoites are purified, counted, and, at a specified concentration, cryopreserved. Cryopreservation commences with the addition of cryoprotective additives to the purified sporozoites to produce the PfSPZ Challenge product. PfSPZ Challenge is dispensed into screw-cap vials containing 15,000 or 50,000 sporozoites in a 20 µL aliquot. PfSPZ Challenge is stored in liquid nitrogen vapour phase at -140°C to -196°C. Each vaccine lot undergoes comprehensive quality control analysis to ensure that the purity, identity and integrity of the virus met pre-defined specifications.

Immediately prior to use, PfSPZ Challenge in cryovials was thawed individually by partial submersion of the vials for 30 seconds in a 37°C ± 1°C water bath. Designated, trained study staff then prepared, diluted (using Phosphate buffered saline (PBS) and 25% Human Albumin Solution (HAS)) and dispensed PfSPZ Challenge to clinical staff at the site. The maximum time interval allowed between thawing of PfSPZ Challenge and administration to a volunteer was 30 minutes. If PfSPZ Challenge had not been administered within this time, the preparation was discarded and a fresh vial of PfSPZ Challenge was thawed and prepared for administration.

**Administration of PfSPZ Challenge**

Volunteers were reviewed in clinic the day before CHMI (C-1) to check on-going eligibility and blood drawn for safety assessment and baseline immunology. PfSPZ Challenge was administered using a needle and syringe either intramuscularly or intradermally. Intradermal injections were administered using the BD Integra 1ml TB syringe & needle (1ml 26G 3/8 (0.45mm x 10mm) Ref 305279). Intramuscular injections were administered using the BD 1ml syringe (Ref 309628) and BD Microlance 3 (23G 1”, 0.6 x 25mm Ref 300800). Study staff administrating PfSPZ Challenge wore gloves and eye protection. During administration of PfSPZ Challenge, advanced life support drugs and resuscitation equipment were immediately available for the management of anaphylaxis. The inoculation sites were immediately covered with a dressing to absorb any PfSPZ Challenge that may have leaked out through the needle track. The dressing was removed from the injection site after 1 hour when the volunteers were reviewed and provided with a thermometer and an emergency contact card detailing the 24 hour telephone number of the on-call study clinician and the sensitivities of the infecting strain of malaria.

**Parasite Growth Modelling**

Quantitative real-time PCR (qPCR) was conducted as previously described (*Ewer et al.* *submitted. Sheehy et al. submitted*). Individuals’ weights were recorded to allow estimation of blood volume. Based upon results obtained using dilution series of microscopically-counted cultured parasites, this method has a lower limit of quantification (LLQ, defined as %CV<20%) of around 20 parasites/ml blood p/ml (*Douglas et al, manuscript in preparation*). Counted parasite dilution series results suggest that the lower limit of probable detection (LLD, i.e. a probability of >50% of ≥1 positive result among three replicate PCR reactions) is in the region of 5 p/ml, while samples at 1 p/ml are consistently negative (24/24 PCR reactions). Positive results in this assay (even at very low level) are thus essentially 100% specific for genuine parasitemia, with positive results beneath the LLQ likely to signify parasitemia in the range 2-20p/ml.

The number of parasites released from the liver (liver-to-blood inoculum, LBI) was estimated in three ways:

A. Maximum qPCR value in first asexual lifecycle (prior to day 8.25) for each volunteer.

B. Linear regression-fitted parasite density at day = 7.5 (*Douglas et al, manuscript in preparation*).

C. LBI estimated derived from sine wave model-fitted parameters 'a' and 'c': 10a+c .

PMR was estimated in two ways:

1. PMR, derived from linear model-fitted parameter ‘m’: 10^2m^
2. PMR derived from sine wave model-fitted parameter ‘m’: 10^2m^

For fitting of linear and sine models, each subject’s results prior to the first point >20p/ml were treated as missing. All subsequent points <20p/ml or negative were replaced with 10p/ml. Modelling was conducted using Stata version 11 (StataCorp, Texas).

**SUPPLEMENTARY REFERENCES**

1. Bejon P, Andrews L, Andersen RF, Dunachie S, Webster D, Walther M, et al. Calculation of liver-to-blood inocula, parasite growth rates, and preerythrocytic vaccine efficacy, from serial quantitative polymerase chain reaction studies of volunteers challenged with malaria sporozoites. The Journal of infectious diseases. 2005;191(4):619-26. Epub 2005/01/19.

2. Simpson JA, Aarons L, Collins WE, Jeffery GM, White NJ. Population dynamics of untreated Plasmodium falciparum malaria within the adult human host during the expansion phase of the infection. Parasitology. 2002;124(Pt 3):247-63. Epub 2002/04/02.
