# Supplementary material for: Optimising Controlled Human Malaria Infection Studies Using Cryopreserved P. falciparum Parasites Administered by Needle and Syringe
Source: PLoS One. 2013 Jun 18;8(6):e65960. doi: 10.1371/journal.pone.0065960 (PMC3688861; doi:10.1371/journal.pone.0065960)
Supplement: Protocol S1 — Study protocol. (PDF) [file pone.0065960.s012.pdf]

Study Reference: VAC 049

PROTOCOL Version 3.1

26th September 2011

A pilot study to optimise controlled human malaria infections using *Plasmodium falciparum* sporozoites administered by needle and syringe

Trial Sponsor: University of Oxford

IND Sponsor: Sanaria*

Chief Investigator: Professor Adrian V.S. Hill

*Sanaria is the Sponsor of the Investigational New Drug (IND) application (IND) for the product to be administered in this trial (PfSPZ Challenge); this IND (IND 14267) is currently filed with the US Food and Drug Administration (FDA)

Modification History

Version	Date	Author(s)
1.0	4th August 2011	Susanne Sheehy, Alison Lawrie, Merryn Voysey, Adrian Hill.
2.0	12th September 2011	Alison Lawrie and Susanne Sheehy
3.0	21st September 2011	Susanne Sheehy & Adrian Hill
3.1	26th September 2011	Susanne Sheehy, Alison Lawrie, Adrian Hill.

A pilot study to optimise controlled human malaria infections using *Plasmodium falciparum* sporozoites administered by needle and syringe.

Study Code: VAC049

**Chief Investigator**	Professor Adrian V.S. Hill<br>Centre for Clinical Vaccinology and Tropical Medicine<br>Churchill Hospital, Old Road, Headington<br>Oxford, OX3 7LJ<br>Tel: 01865 857417<br>Fax: 01865 857471<br>Email: *adrian.hill@ndm.ox.ac.uk*
**IND Sponsor**	Dr Stephen L. Hoffman<br>Sanaria Inc.<br>9800 Medical Center Drive, Suite A209<br>Rockville, MD 20850<br>E-mail:slhoffman@sanaria.com
**Trial Site**	Centre for Clinical Vaccinology and Tropical Medicine<br>University of Oxford<br>Churchill Hospital, Old Road, Headington,<br>Oxford, OX3 7LJ<br>*Principal Investigator at Site: Professor Adrian Hill*
**Sponsoring Institution**	University of Oxford<br>Clinical Trials and Research Governance<br>Joint Research Office, Block 60, Churchill Hospital,<br>Old Road, Headington, Oxford, OX3 7LJ<br>Tel: 01865 572245<br>Fax: 01865 572228<br>Email: heather.house@admin.ox.ac.uk
**Local Safety Monitor**	Dr Brian Angus<br>Centre for Clinical Vaccinology and Tropical Medicine<br>Churchill Hospital, Old Road, Headington<br>Oxford, OX3 7LJ<br>Tel: 01865 553 20289<br>Email: brian.angus@ndm.ox.ac.uk

Monitor

Clinical Trials and Research Governance
Joint Research Office, Block 60, Churchill Hospital,
Old Road, Headington, Oxford, OX3 7LJ
Tel: 01865 572245
Fax: 01865 572228
Email: heather.house@admin.ox.ac.uk

Investigator Agreement

"I have read this protocol and agree to abide by all provisions set forth therein.

I agree to comply with the principles of the International Conference on Harmonisation Tripartite Guideline on Good Clinical Practice."

------------------------------------	------------------------------------	------------------------
Chief Investigator	Investigator Signature	Date
Professor Adrian Hill		

Confidentiality Statement

This document contains confidential information that must not be disclosed to anyone other than the trial Sponsor, the IND sponsor, the Investigator Team, and members of the Independent Ethics Committee. This information cannot be used for any purpose other than the evaluation or conduct of the clinical investigation without the prior written consent of Professor Adrian Hill.

TABLE OF CONTENTS

1. SYNOPSIS 7
2. ABBREVIATIONS 8
3. BACKGROUND AND RATIONALE 9
4. Study overview 21
5. INVESTIGATIONAL PRODUCT – PfSPZ Challenge 24
6. RECRUITMENT AND WITHDRAWAL OF TRIAL VOLUNTEERS 26
7. TREATMENT OF TRIAL VOLUNTEERS 30
8. ASSESSMENT OF SCIENTIFIC OBJECTIVES 39
9. ASSESSMENT OF SAFETY 40
10. STATISTICS 46
11. QUALITY CONTROL AND QUALITY ASSURANCE PROCEDURES 47
12. ETHICS 48
13. DATA HANDLING AND RECORD KEEPING 49
14. FINANCING AND INSURANCE 50
15. Appendix A. Laboratory values for exclusion 51
16. references 52

1. SYNOPSIS

**Title**	**A pilot study to optimise controlled human malaria infections in humans using *Plasmodium falciparum* sporozoites administered by needle and syringe.**
**Trial Centre**	Centre for Clinical Vaccinology and Tropical Medicine (CCVTM)<br>Old Road, Headington, Oxford, OX3 7LJ, UK
**Trial Identifier**	VAC 049
**Design**	Open label pilot study
**Population**	Healthy adults aged 18 – 45 years
**Sample Size**	**Group 1 (n=6)**<br>2,500 sporozoites administered intradermally in 2 sites (one injection of 50µl containing 1,250 sporozoites in each deltoid).<br><br>**Group 2 (n=6)**<br>2,500 sporozoites administered intramuscularly in 2 sites (one injection of 50µl containing 1,250 sporozoites in each deltoid).<br><br>**Group 3 (n=6)**<br>25,000 sporozoites administered intramuscularly in 2 sites (one injection of 50µl containing 12,500 sporozoites in each deltoid).<br><br>**Total: 18 volunteers**
**Follow-up duration**	3 months
**Planned Trial Period**	4 months
**Primary Objective**	To determine the infectivity rates of PfSPZ Challenge administered in various regimens.
**Secondary Objective**	To assess the safety of PfSPZ Challenge administered in various regimens.
**Tertiary Objectives**	To determine the dynamics of *Plasmodium falciparum* parasite growth following PfSPZ Challenge administered in various regimens.
**Investigational Product**	Aseptic, purified, cryopreserved *Plasmodium falciparum* sporozoites for challenge (PfSPZ Challenge).
**Form**	Liquid
**Route of Administration**	Intradermal (ID) or intramuscular (IM) needle injection in the deltoid region of the arm(s).
**Dose per Administration**	2,500 or 25,000 *Plasmodium falciparum* sporozoites.

2. ABBREVIATIONS

**AE**	Adverse event
**BSC**	Biological Safety Cabinet
**CCVTM**	Centre for Clinical Vaccinology and Tropical Medicine,
**CHMI**	Controlled Human Malaria Infection
**CRF**	Case Report Form or Clinical Research Facility
**CTRG**	Clinical Trials and Research Governance – The University of Oxford
**FBC**	Full blood count
**FDA**	US Food and Drug Administration
**GCP**	Good Clinical Practice
**GMP**	Good Manufacturing Practice
**GP**	General Practioner
**HBsAg**	Hepatitis B Surface Antigen
**HCG**	Human Chorionic Gonadotrophin
**HCV**	Hepatitis C virus
**HIV**	Human immunodeficiency virus
**HSA**	Human serum albumin
**IMP**	Investigational Medicinal Product
**IND**	Investigational New Drug
**LSM**	Local safety monitor
**MHRA**	Medicines and Healthcare products Regulatory Agency
**NHS**	National Health Service
**PBS**	Phosphate buffered saline
**PCR**	Polymerase chain reaction
**P. falciparum**	*Plasmodium falciparum*
**PMR**	Parasite Multiplication Rate
**PfSPZ Challenge**	Aseptic, purified, cryopreserved *P. falciparum* sporozoites
**R&D**	Research & Development
**REC**	Research Ethics Committee
**RUNMC**	Radboud University Nijmegen Medical Centre
**SAE**	Serious adverse event
**SOP**	Standard Operating Procedure
**SmPC**	Summary of Product Characteristics
**SUSAR**	Suspected unexpected serious adverse reaction
**USMMVP**	United States Military Malaria Vaccine Programme
**WHO**	World Health Organisation

3. BACKGROUND AND RATIONALE

Epidemiology of Malaria

Although recent and encouraging evidence suggests that the epidemiology of *Plasmodium falciparum* malaria is changing across certain parts of Africa,[1] the worldwide burden of disease from malaria remains a major public health problem, with approximately 250 million cases worldwide in 2008, mostly in Africa.[2] The enormous economic and social consequences of malaria have been well documented.[3]

The development of resistance both in Anopheles mosquitoes to certain insecticides and of malaria parasites to chemotherapeutic agents has contributed to an increasing need for new, effective interventions for the prevention or treatment of malaria.[4]

To provide a coordinated global approach to fighting malaria, the Roll Back Malaria (RBM) Partnership was launched in 1998 by the World Health Organization (WHO), the United Nations Children's Fund (UNICEF), the United Nations Development Programme (UNDP) and the World Bank.[5]

Lifecycle of the malaria parasite

The lifecycle of the parasites that cause malaria in humans is complex with stages in both human and mosquito hosts (Figure 1). The bites of infected female Anopheles mosquitoes transmit malaria sporozoites to the human host where they travel via the bloodstream to the liver and invade hepatocytes (*liver stage*). Here they mature into merozoites for 6 to 7 days after which the hepatocytes rupture releasing a large number of merozoites into the bloodstream. Merozoites then invade erythrocytes where they multiply and after 2 days cause the erythrocyte to rupture, releasing progeny merozoites that in turn invade new erythrocytes (*blood stage*). A small percentage of merozoites differentiate into gametocytes, which when ingested by a mosquito, unite with another gametocyte to create a zygote. The zygote matures and releases sporozoites, which migrate to the mosquito's salivary glands and are injected into the human when the mosquito feeds. Infection by sporozoites and the liver-stage of malaria is asymptomatic. It is the blood stage of infection that is associated with symptoms and potentially severe or fatal complications.

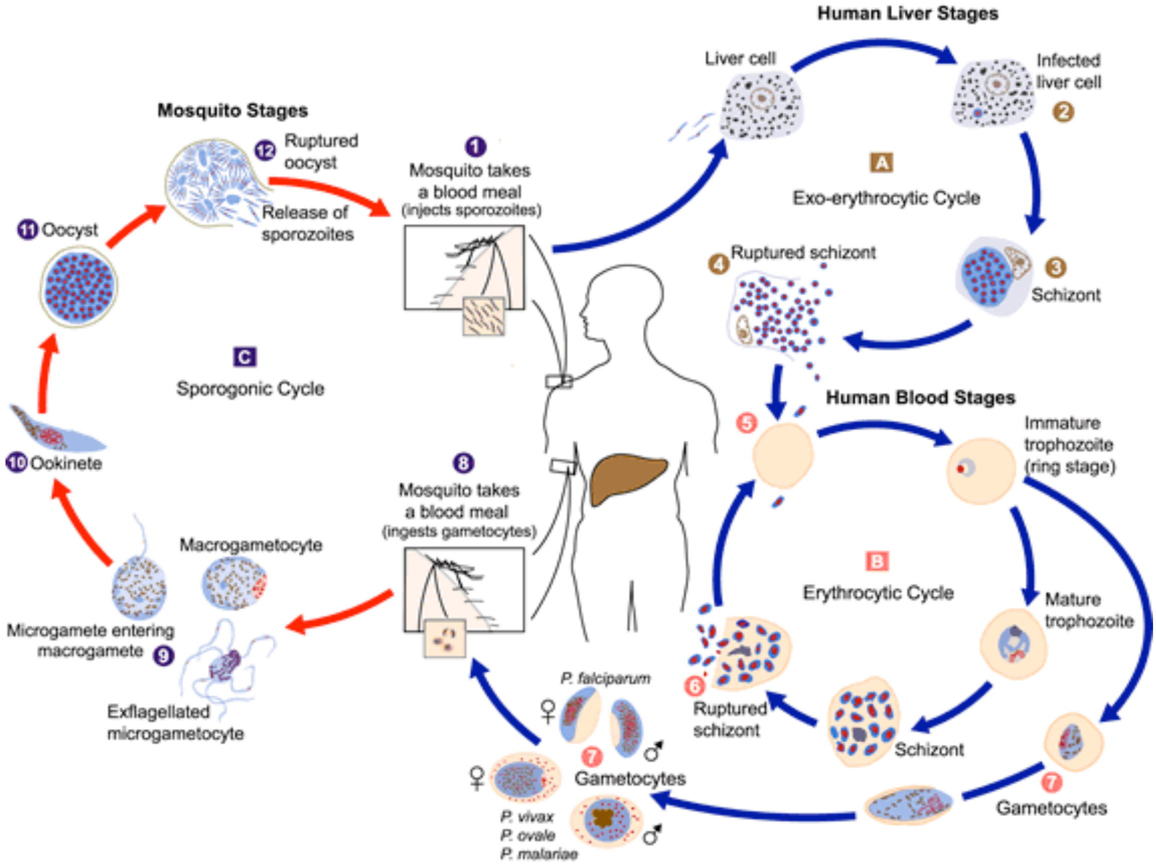

Figure 1 *Lifecycle of Malaria*

Microbial Challenge Studies of Human Volunteers

The deliberate infection of human volunteers with micro-organisms have contributed uniquely to our understanding of the pathogenesis, immune responses and the treatment and prevention of numerous microbial diseases including influenza, cholera, typhoid and hepatitis.[6] A review by the UK Academy of Medical Sciences on microbial challenge studies recognised that such studies are essential for providing proof of concept for therapeutic interventions and can significantly accelerate progress to Phase III studies.[6]

Controlled Human Malaria Infection (Challenge)

Malaria is a microbe particularly well suited to challenge studies. It has a relatively short asymptomatic period, a well-established diagnostic laboratory test (thick film microscopy), and no long term sequelae or infectious state following appropriate and timely treatment. Studies involving controlled human malaria infection (CHMI) are a powerful tool for investigating malaria vaccine and prophylactic drug efficacy.[7] With an increasing number of candidate malaria vaccines being developed, the number of centres conducting CHMI is expanding to increase the testing capacity worldwide.[7]

Deliberate infection of humans with malaria was first performed in 1917 by Wagner von Jauregg, primarily as a therapy for patients with neurosyphilis.[8] Thousands of patients underwent the treatment (the objective of which was to induce a febrile illness that was thought beneficial for the progress of the disease), administered by the bites of infectious mosquitoes or by intravenous or subcutaneous inoculation of dissected *Plasmodium* sporozoites suspended in media. The practice stopped with the advent of antibiotics.

In the 1960s, CHMI trials were used to assess the effects of anti-malaria treatments on healthy non-immune male inmates in the United States.[9] Following the development of protocols for the continuous culture of *P. falciparum* in 1976[10] and for the generation of mature *P. falciparum* gametocytes *in-vitro* in 1981,[11] it became possible to produce laboratory-reared infectious mosquitoes,[12] meaning that CHMI trials could be performed more routinely.

The first well-documented CHMI with laboratory-reared infectious mosquitoes was carried out in 1986 at the US Walter Reed Army Institute of Research (WRAIR), the US Naval Medical Research Institute (NMRI) and the US National Institutes of Health (NIH). Six volunteers were infected with *P. falciparum* sporozoites by the bites of infectious Anopheles freeborni and Anopheles stephensi mosquitoes.[13] The following year, the efficacies of the first recombinant protein and synthetic peptide *P. falciparum* vaccines were tested in experimentally infected volunteers.[14,15]

CHMI has now become established as a key tool to assess the efficacy of novel malaria vaccines and drugs.[7] As CHMI trials are carried out in a controlled environment, they allow unprecedented detailed evaluation of parasite growth and immunological responses, providing essential information for vaccine and drug development.[7]

Since the late 1980s, the number of institutions carrying out CHMI with *P. falciparum* has been growing. In 2007, data were published from a total of 532 volunteers.[16] So far, unpublished analysis shows that a total of 1,343 volunteers were experimentally infected with *P. falciparum* between 1985 and 2009.[17]

Conduct of CHMI Trials

Following a collaborative consensus process involving investigators from the USMMVP, Sanaria, University of Maryland, University of Oxford, RUNMC, The Seattle Biomedical Research Institute and the KEMRI-Wellcome Kilifi Research Programme, a consensus document; *"Standardization of Design and Conduct of P. falciparum Sporozoite Challenge Trials"* was developed, and provides a comprehensive guide to the appropriate conduct of malaria challenge studies.[18] Although there remain minor differences between centres in follow-up procedures in CHMI trial conduct, there is consensus on the following key points.

- All volunteers should have a medical assessment no longer than 48 hours before challenge, including an interim medical history, directed physical examination, pregnancy test for female volunteers.
- Follow-up visits should be scheduled at least once daily, but may increase in frequency to two or three times daily, starting at day 5-7 post-challenge. At all visits volunteers should be questioned about the occurrence of adverse events and use of medication.
- In the event that a volunteer does not attend for a scheduled follow-up visit it is imperative that investigators find that volunteer as quickly as possible and assess them for patent parasitaemia and clinical malaria. Should the volunteer withdraw consent from further follow-up prior to receipt of antimalarial drugs, it may be appropriate to withdraw the volunteer from the trial protocol and administer a course of antimalarial chemotherapy under close supervision.
- Grading and reporting of adverse events should be performed using international and local guidelines. It should be noted that the occurrence of a low frequency of grade 3 severe adverse events, of short duration, and with no long-term sequelae, is not unexpected in clinical challenge trials. A minority of those challenged are known to experience grade 3 systemic adverse events and this fact should be included in the informed consent form.
- Vital signs should be recorded at least once daily and at any subsequent visits for medical attention. Directed physical examination should be performed when necessary.
- It is critical that every volunteer must receive every dose of anti-malarial therapy. In some settings fully directly observed treatment will be essential. Where directly observed treatment is not used, investigators must follow volunteers closely to ensure compliance with the treatment regimen.
- After challenge, all volunteers should be followed until they have completely finished anti-malaria treatment.
- Volunteers should be evaluated at least two weeks after finishing treatment.
- A local safety monitor and an independent safety monitoring committee should be established to act as independent experts in evaluating adverse events. The safety monitor or monitoring committee may advise the investigators on initiating anti-malarial treatment for a specific volunteer or volunteer group. While safety monitoring committees are not a requirement for Phase 1 trials, they should be considered a requirement for malaria challenge trials which have an efficacy component and which have major potential safety concerns.

Clinical Presentation Post CHMI

Nearly all volunteers in malaria challenge studies develop symptoms of clinical malaria infection; approximately one-fifth of volunteers temporarily develop symptoms graded as severe (symptoms that prevent daily activities), but severe or life-threatening malaria has never occurred.[19] The most common symptoms are fatigue and headache, and severe symptoms can include headache, fatigue, malaise, chills, myalgia, rigors, nausea and vomiting. Clinical symptoms generally coincide with the detection of blood-stage parasites at densities of 10–20 parasites per µl of blood by microscopy of thick film microscopy smears.[19] This corresponds to a parasitaemia of approximately 0.0004%.[7] Severe malaria is generally diagnosed when parasitaemia is 3 to 4 logs greater than the peak parasitaemia in challenge trials. After the start of malaria treatment, symptoms can temporarily increase in severity but subside quickly with an average duration of approximately 2–3 days.[7]

Routine laboratory checks generally show a moderate decrease in leukocyte and platelet numbers during infection, with no change in haemoglobin concentration.[20] Bleeding or thrombogenic complications have never been described.[19,20] Abnormalities of liver enzymes have been observed, but these abnormalities did not result in clinical manifestations and they resolved after a few days.[19,20]

Immediate treatment of volunteers at the earliest phase of microscopically detectable blood-stage infection ensures that the potential risks of complications associated with severe malaria are minimized to the greatest extent possible. Indeed, human malaria challenge infections have been shown to be safe in the 1,343 volunteers challenged so far.[16,19,20] Recently, safety concerns were raised because of a cardiac event in a young volunteer shortly after treatment for diagnosed malaria, although a definite relationship between the cardiac event and the experimental malaria infection was not established.[21] Nevertheless, it has been generally agreed that volunteers with an increased risk of cardiac disease should be excluded from such trials.[18]

Ethical Considerations of CHMI trials

For any clinical trial, the risk for potential volunteers should be weighed against the benefit. As, often no direct benefit accrues to challenge trial participants, benefits are considered in the context of possible public health gains that may occur as a result of scientific advances made through malaria challenges. This places a burden on the challenge trial investigators both to exercise all possible safeguards for volunteer safety (primary consideration) and to ensure that maximal scientific benefit accrues from each challenge trial (secondary consideration). Key ethical considerations agreed by consensus of the field include;[18]

1. Safety is the paramount consideration in conduct of malaria challenge trials. When challenge trials are conducted at existing and new centres, the practical considerations should always be focused on volunteer safety.
2. Investigators are required to follow both international and local guidelines with respect to ethical considerations and in accordance with the Declaration of Helsinki and should fulfil all local regulatory and ethics committee requirements.
3. Malaria Challenge trials should be conducted according to ICH and/or WHO Good Clinical Practice Guidelines. The scientific benefit should be maximized whilst minimizing risk and discomfort/distress to individuals. From this perspective it is important that the results of challenge trials of candidate malaria vaccines enable comparative evaluation and collect as much information as is reasonably possible about the impact of the vaccine on *P. falciparum,* both in terms of the load of merozoites that emerges from the liver ("the liver-to-blood inoculum") and the parasite growth rate subsequently prior to initiation of drug treatment as well as the proportion of volunteers who are completely protected. The availability of data to the scientific community also attains an ethical dimension in this perspective, with importance attached to access to data that may inform design of future challenge trials and design of malaria vaccines.
4. The raw data (both microscopy and PCR where available) from challenge trial datasets should be made publicly available to facilitate scientific benefit to the community.
5. If an unexpected SAE which is possibly related to malaria challenge occurs at a challenge trial centre, recognizing legal restrictions, every effort should be made to communicate information on this SAE to the community of challenge trial centres within 90 days of the occurrence of the SAE. This is in addition to the usual reporting requirements to ethical committees, sponsors (trial and IND sponsors) and regulatory authorities. SAEs which are unambiguously not related to the challenge procedure are excluded, e.g. hospitalizations for clearly coincidental events such as trauma. Where there is any doubt community-wide notification should occur. This is because safety of participants at other centres may be affected by occurrence of an SAE at one centre.

Oxford's Experience Conducting CHMI trials

The University of Oxford have been conducting experimental malaria infections for the last 11 years. To date, more than 300 volunteers have undergone experimental malaria challenge at this site, including more than 80 unvaccinated control volunteers (Table 1). A recent meta-analysis has shown that the symptoms of malaria experienced by volunteers undergoing sporozoite challenge by mosquito bite in Oxford are broadly similar to those experienced by volunteers challenged by mosquito bite at RUNMC and the USMMVP (Roestenberg *et al. manuscript in preparation*).

In Oxford, three volunteers have required admission to hospital for observation following challenge (SAE related to challenge).

- Patient 1: VAC013: Vaccinated Volunteer: Admitted for 48 hours due to vomiting and received intra-venous fluids and anti-emetics as additional therapy.
- Patient 2: VAC022: Vaccinated Volunteer: Admitted for 12 hours following the development of mild eyelid oedema following chloroquine treatment. This resolved spontaneously and was considered related to the treatment with chloroquine.
- Patient 3: VAC039: Unvaccinated Control volunteer: Admitted to hospital for 24 hours for intravenous fluid rehydration and observation following a pre-syncopal episode and moderately severe symptoms of uncomplicated malaria.

Trial	Year	Type of Challenge	No. Vaccinees Challenged	No. Controls Challenged	No. SAEs Related to Challenge
VAC004	2000	Sporozoite	12	3 (6)	0
VAC007	2000	Sporozoite	9	3	0
VAC013	2001	Sporozoite	14	11	1
VAC015[22]	2001/3	Sporozoite	16	5	0
VAC017[22]	2002	Sporozoite	16	4	0
VAC018[23]	2002/3	Sporozoite	5	5	0
VAC021[24]	2003/4	Sporozoite	16	6	0
VAC022[25]	2003	Sporozoite	11	6	1
VAC023[26]	2003/4	Sporozoite	15	6	0
VAC027[27]	2006/7	Sporozoite	15	6	0
VAC030[28]	2005/6	Sporozoite	24	6	0
MAL034	2009/10	Sporozoite	43	12	0
VAC035[29]	2010	Blood	5	3	0
VAC039	2010	Sporozoite	36	6	1
BSV 1[30]	2005	Blood	0	6	0
TOTAL			237	88	3

Table 1: *CHMI trials conducted to date by the University of Oxford at CCVTM. All volunteers were malaria naïve. Sporozoite = mosquito bite. Blood = Blood stage challenge. Control = unvaccinated infectivity controls.*

In 2009, in a Phase I/IIa sporozoite challenge study assessing the efficacy of viral vectored malaria vaccines in Oxford (VAC039; Clinical trials.gov reference: NCT01142765), one volunteer who underwent sporozoite challenge on 1st October 2010 failed to attend his next scheduled study visit on 7th October 2010. The police were immediately informed and began a nationwide search for the individual. All volunteers had been informed at screening that the police would be notified should they go missing following challenge, if they had not completed a full course of an appropriate anti-malarial treatment. The volunteer was found in The Netherlands by the local police 17 days following challenge. He then had very mild malaria symptoms. He was admitted to a local hospital where he received appropriate treatment for *P. falciparum.* He had no signs of severe malaria but showed an altered mental state considered unrelated to malaria with apparent memory loss and suicidal ideation. He was therefore transferred for in-patient psychiatric assessment and discharged a few days later to his GP's care.

It emerged that from 2nd October 2010, the day after challenge, the volunteer had experienced an alteration in his expected behaviour following his arrest the previous evening by the police relating to their investigation of a serious crime. This arrest appeared to trigger his leaving home and disappearance and appears relevant to the subsequent finding of memory loss and suicidal ideation. It emerged on subsequent investigation that the volunteer actually had a history of some

psychiatric morbidity pre-dating his involvement in the study by many years, which was not disclosed at screening by the volunteer or his general practitioner. Of note, the volunteer had attended 9 clinic visits prior to challenge and appeared a reliable and appropriate volunteer.

This event was extensively discussed with investigators, colleagues and appropriate authorities and a non-study related causality agreed. Given the exceptional circumstances relating to this case, it seems very unlikely that a similar event would happen again in the future. Management of the event was extensively reviewed by the trial's sponsor, the MHRA and REC who felt that appropriate and timely action was taken. Follow-up procedures following malaria challenge have been reviewed locally, and it has been decided that for future studies volunteers should be contacted daily on days 1-5 post challenge in order to make sure they are contactable and well.

Methods of Inducing *P. falciparum* Infection for CHMI Trials

Currently, there are three methods of performing experimental human malaria infections;

1. Experimental blood stage infection

A small, known number of erythrocytes infected with *P. falciparum* merozoites is administered intravenously in a saline solution to volunteers. The number of parasites inoculated is approximately 1,000 times lower than the estimated number of merozoites released from the liver following a standard experimental sporozoite challenge with bites from five infected mosquitoes. This allows for an extended blood-stage follow-up of approximately three more replication cycles (6 days) before thick film microscopy detection thresholds are reached, with obligatory treatment.[7]

A master cell bank of infected erythrocytes for human clinical use has been generated by storing infected erythrocytes from two parasitaemic volunteers who were infected by mosquito bites, in compliance with blood bank safety criteria.[31] Since the 1990s, approximately 50 humans have been infected by direct inoculation of infected erythrocytes from this master cell bank.[7] The length of the prepatent period (the interval from inoculation until infected erythrocytes are microscopically detectable) correlates with the number of inoculated parasites.[9] With inoculae as small as 300 infected erythrocytes, parasite growth curves were generated over a 7–9-day period before initiation of treatment was required.[31]

The blood-stage challenge model has several potential shortcomings;

- The viability of the injected parasites can only be determined retrospectively by culture, so it is difficult to standardize the exact number of viable injected parasites. Differences of a factor of ten in terms of the number of viable parasites have been described between inoculae.[30,32]
- The small number of inoculated parasites allows for a long window of observation, but may also boost the immune response.[33]
- The liver stage of parasite development is circumvented by this model, bypassing potential immune effects induced by the vaccine on liver-stage parasites.
- The donors of the current master bank of infected erythrocytes were seropositive for both Cytomegalovirus and Epstein barr virus. As a result all volunteers taking part in blood-stage challenges must also be seropositive for both of these viruses, which places a considerable limitation on recruitment in certain populations.[34]

2. Experimental sporozoite infection – Mosquito Bites

The delivery of sporozoite-stage malaria parasites by mosquito bites has traditionally been used as a model to test pre-erythrocytic stage vaccines. Since the late 1980s, standardization of experimental sporozoite infections has improved and efforts to further increase harmonization are ongoing. Such infections are currently routinely carried out at: the US Military Malaria Vaccine Program; the University of Maryland, USA; Radboud University Nijmegen Medical Centre (RUNMC), the Netherlands; the University of Oxford, UK; and, more recently, Seattle Biomed, USA.[17] All centres use *A. stephensi* mosquitoes that feed on either the chloroquine-sensitive NF54

strain of *P. falciparum* or the 3D7 clone of NF54. In addition, limited numbers of volunteers have been challenged with the 7G8 strain of P. falciparum.[16]

Approximately 14–21 days after feeding, mosquitoes are checked for infection by microscopic examination of salivary glands. Healthy human volunteers are subsequently exposed to the bites of five infectious mosquitoes for either 5 or 10 minutes. Almost 100% of volunteers bitten by five infected mosquitoes develop patent parasitaemia, with very rare exceptions.[19,20] Infection rates drop significantly when volunteers are exposed to fewer than five infected mosquitoes.[20,35]

A major strength of the sporozoite infection model is the use of infectious mosquitoes, mimicking the natural route of infection. However, the mosquito bite challenge model is limited by a number of factors;

- It is impossible to control the number of sporozoites inoculated by biting mosquitoes. This number is generally thought to vary up to a maximum of several thousand sporozoites.[7]
- The challenge can only be performed in centres with access to an appropriate insectary and entomology staff, considerably limiting the number of sites internationally that can perform these trials.[7]

3. Experimental sporozoite infection using aseptic, purified, cryopreserved sporozoites

In principle, the most accurate and practical way of dosing sporozoites is to inject them directly by needle and syringe.[7] This would have the following advantages over the traditional mosquito bite sporozoite challenge;

- It would allow experimental challenge trials to be conducted at multiple sites that currently do not have access to *P. falciparum* sporozoite-infected mosquitoes.
- The number of sporozoites with which volunteers are inoculated could be easily calculated and predefined.
- The potential impact of variation in infectivity of mosquito-delivered sporozoites when performing parallel clinical trials at multiple sites or sequential clinical trials at the same site would be eliminated.
- Practical advantages, including ease of administration and the ability to challenge of volunteers over an extended period rather than all on the same day.

Sanaria Inc. is a biotechnology company that has developed aseptic, purified cryopreserved *P. falciparum* sporozoites (NF54 strain) for CHMI via injection (PfSPZ Challenge). The salivary glands of aseptic *A. stephensi* mosquitoes infected with *P. falciparum* sporozoites are removed by dissection and triturated to release the sporozoites. The sporozoites are purified, counted and cryopreserved at a specified concentration to produce the challenge inoculum; PfSPZ Challenge. This process is in compliance with current Good Manufacturing Practices (cGMPs) and regulatory requirements for production of a high-quality PfSPZ Challenge.

The first CHMI trial using aseptic cryopreserved *P. falciparum* sporozoites administered by needle injection was performed by RUNMC in 2010/11 (Clinical Trials.gov: NCT01086917 Table 2). This was a dose finding study to establish which dose of PfSPZ Challenge administered intradermally would infect 100% of inoculated volunteers.

Group Number	No. of volunteers	Total Dose of PfSPZ	Route of Administration	Number of injection sites	No. of Volunteers Infected
1	6	2,500 sporozoites*	Intradermal	Two	5/6 (83%)
2	6	10,000 sporozoites*	Intradermal	Two	5/6 (83%)
3	6	25,000 sporozoites*	Intradermal	Two	5/6 (83%)

Table 2: *Overview of trial groups RUNMC trial of PfSPZ Challenge. *Total dose of PfSPZ split between two administration sites.*

Inoculation of PfSPZ Challenge was well tolerated, associated with no local or systemic AEs. In all 3 groups, 5 out of 6 inoculated volunteers were successfully infected with *P. falciparum* (an infectivity rate of 83%) that was independent of PfSPZ Challenge dose. The first positive blood slides were found between day 11 to 14.3 after administration of PfSPZ Challenge. The geometric mean pre-patent period was similar for all dose groups i.e. 13.0, 12.7, and 13.0 days for the groups receiving 2,500, 10,000, and 25,000 PfSPZ respectively. Comparison with data from this site from CHMI studies using mosquito bites showed that the pre-patent period was longer and parasite multiplication rates higher with PfSPZ Challenge compared to CHMI administered by mosquito bite (R. Sauerwein personal communication). Parasite densities at the time of diagnosis were not significantly different among the groups (12,300, 11,200, and 23,400 parasites/mL in the three groups respectively). Volunteers developed symptoms of *P. falciparum* infection (Table 3 & Figure 2) that were similar to those seen following CHMI administered by mosquito bite at this centre (Roestenberg manuscript in prep).[19]

	2,500 (n=6)		10,000 (n=6)		25,000 (n=6)	
**ANY ADVERSE EVENT**	frequency	mean duration ± SD (days)	frequency	mean duration ± SD (days)	frequency	mean duration ± SD (days)
Abdominal pain	1	2,9±0,0	1	0,0±0,0	2	0,3±0,1
Arthralgia	0	N/A	0	N/A	0	N/A
Chest pain	1	0,04±0,0	0	N/A	0	N/A
Chills	1	2,0±0,0	2	0,3±0,2	2	0,9±0,6
Diarrhea	0	N/A	0	N/A	1	0,8±0,0
Fatigue	5	2,9±3,3	3	2,5±1,7	5	3,0±3,9
Fever	3	1,6±1,5	2	1,8±0,6	4	0,8±0,4
Headache	6	1,1±1,1	6	1,5±1,6	6	1,4±2,6
Malaise	2	2,2±2,4	5	1,8±1,4	1	0,7 ±0,6
Myalgia	2	3,7±3,2	2	1,3±0,5	2	0,8±0,1
Nausea	3	1,7±1,3	5	0,9±0,9	3	1,0±0,9
Vomiting	0	N/A	2	0,0±0,0	0	N/A
**Any**	**6**	**2,0±1,4**	**6**	**1,1±0,8**	**6**	**1,1±1,0**
**GRADE 3 ADVERSE EVENT**						
Abdominal pain	0	N/A	0	N/A	0	N/A
Arthralgia	0	N/A	0	N/A	0	N/A
Chest pain	0	N/A	0	N/A	0	N/A
Chills	0	N/A	0	N/A	0	N/A
Fatigue	0	N/A	0	N/A	1	2,2±0,0
Fever	0	N/A	1	1,2±0,0	0	N/A
Headache	2	3,0±0,4	0	N/A	0	N/A
Malaise	1	4,8±0,0	0	N/A	1	0,1±0,0
Myalgia	0	N/A	0	N/A	0	N/A
Nausea	0	N/A	0	N/A	0	N/A
Vomiting	0	N/A	2	0,0±0,0	0	N/A
**Any**	**2**	**3,9±0,2**	**3**	**0,6±0**	**2**	**1,2±0**

Table 3: *Number and mean duration of solicited AEs possibly or probably related to P. falciparum infection following administration of PfSPZ Challenge*

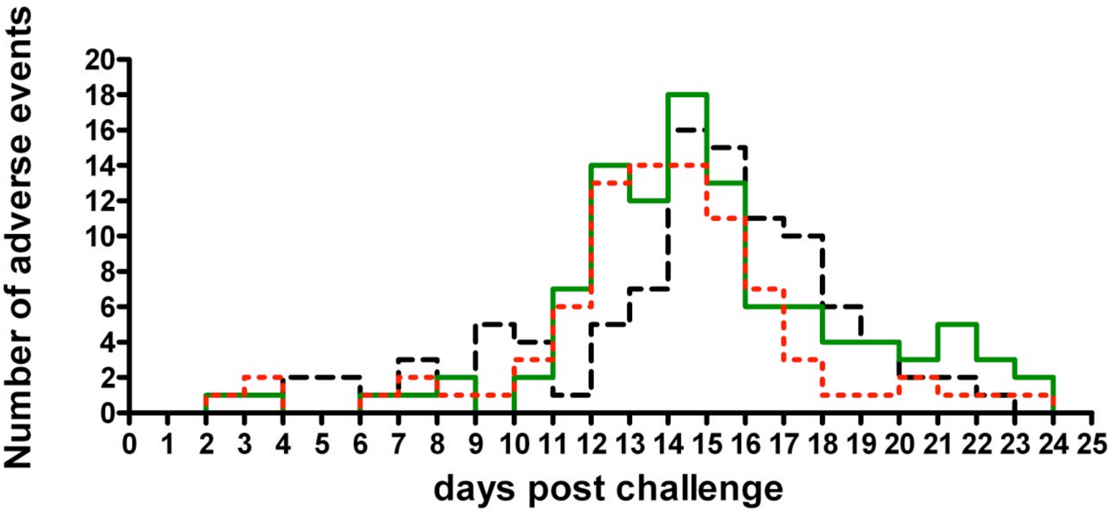

Figure 2: *Number of AEs over time possibly or probably related to P. falciparum infection following administration of PfSPZ Challenge (2,500 (black dashed), 10,000 (red dotted) and 25,000 sporozoites (green straight).*

It is important that any CHMI model infects 100% of control volunteers. Why 100% infectivity was not achieved is this study is unclear. At a meeting sponsored by the European Malaria Vaccine Development Association (EMVDA) in Amsterdam in June 2011, principal investigators from institutions undertaking CHMI studies around the world met to discuss optimal ways to increase infectivity with PfSPZ Challenge.

Murine data presented at this meeting showed that the route of administration of sporozoites was a key determinant of infectivity. C57/BL6 mice (n=5 per group) were injected with 50,000 sporozoites administered by intravenous (200 ul in the tail vain), intramuscular, subcutaneous or intradermal routes (50 ul in each hind leg). Parasite liver load was subsequently determined by *in vivo* imaging as previously described.[36] Results clearly demonstrated that intravenous administration was associated with the greater infectivity (Figures 3 & 4). Intramuscular administration was associated with greater infectivity than subcutaneous or intradermal injection.

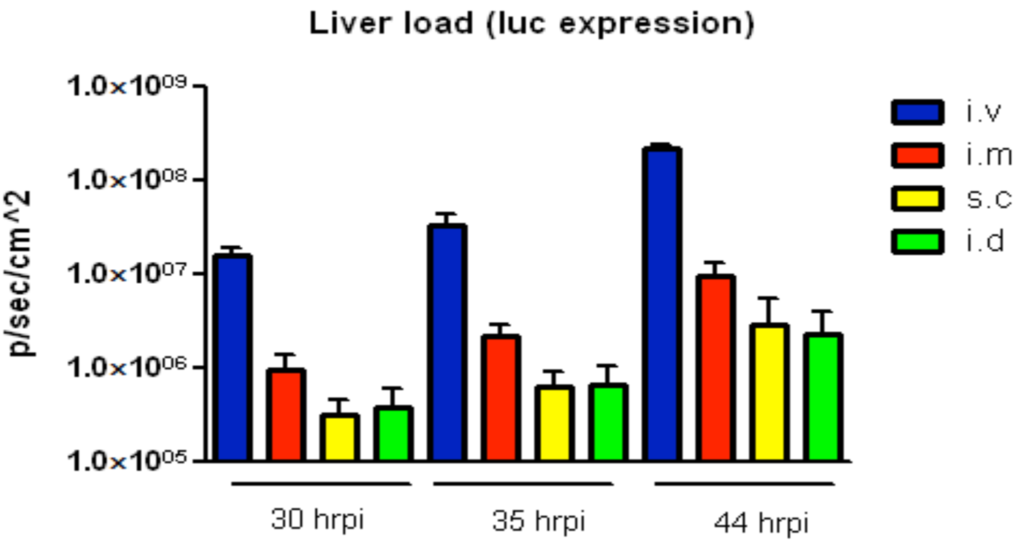

Figure 3: *Liver load of sporozoites in C57BL6 mice 30, 35 and 44 hours post injection. IV= intravenous, IM= intramuscular, SC = subcutaneous, ID = intradermal.*

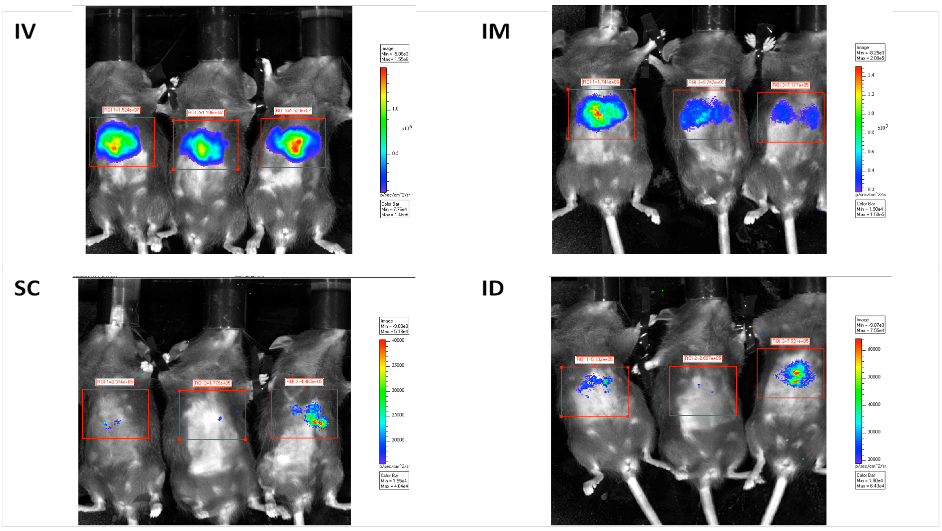

30 hrs post infection

Figure 4: *Liver load of sporozoites in C57BL6 mice 30hours post injection. IV= intravenous, IM= intramuscular, SC = subcutaneous, ID = intradermal.*

Further murine studies using similar *in-vivo* imaging techniques have shown that reducing the volume of injection and increasing the number of injection sites for a given number of sporozoites is associated with increased infectivity rates (*Ploemen et al*. Unpublished data) (Figures 5 and 6).

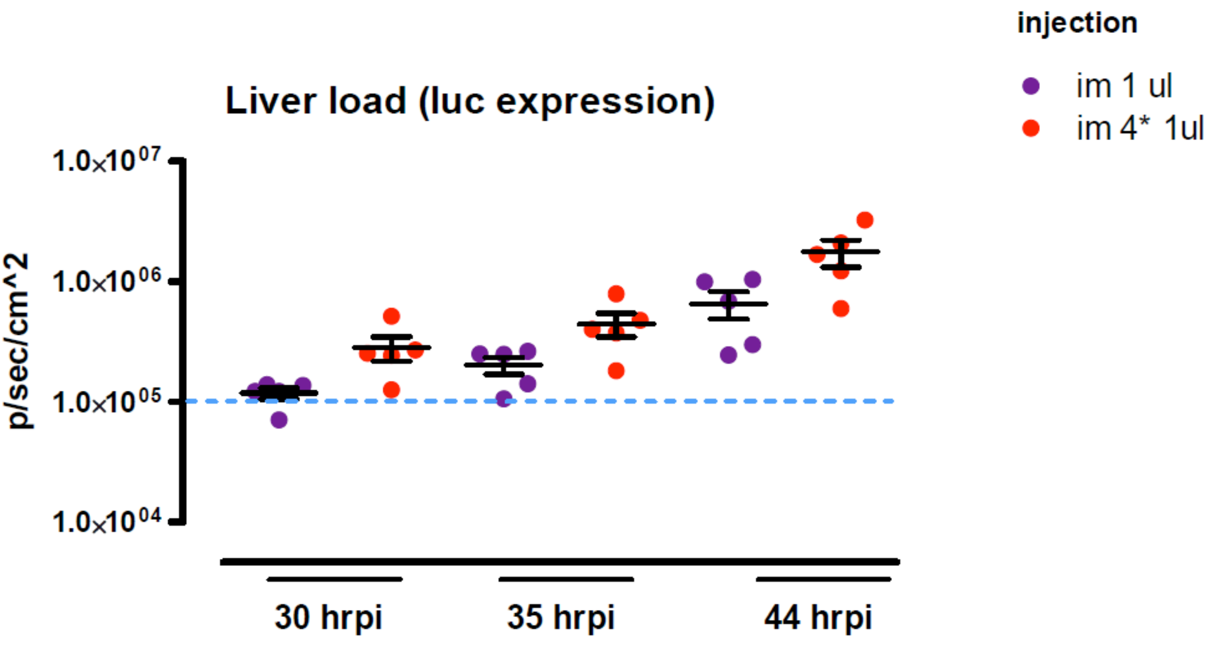

Figure 5: Liver load after intramuscular and intradermal injection; single versus multiple injection sites. *Real time measurement of the liver load in mice injected with 5 x 10^4 sporozoites by intramuscular injection in a bolus or in multiple injections of 1 microlitre volume (n=5 per group) at different time points post injection (30, 35 and 44 hours post infection). Dots represent the luminescent intensity (photon/sec/cm^2) of the regions of interest overlaying the livers from the individual mice. Dotted line represents the threshold of luminescent signalling.*[36]

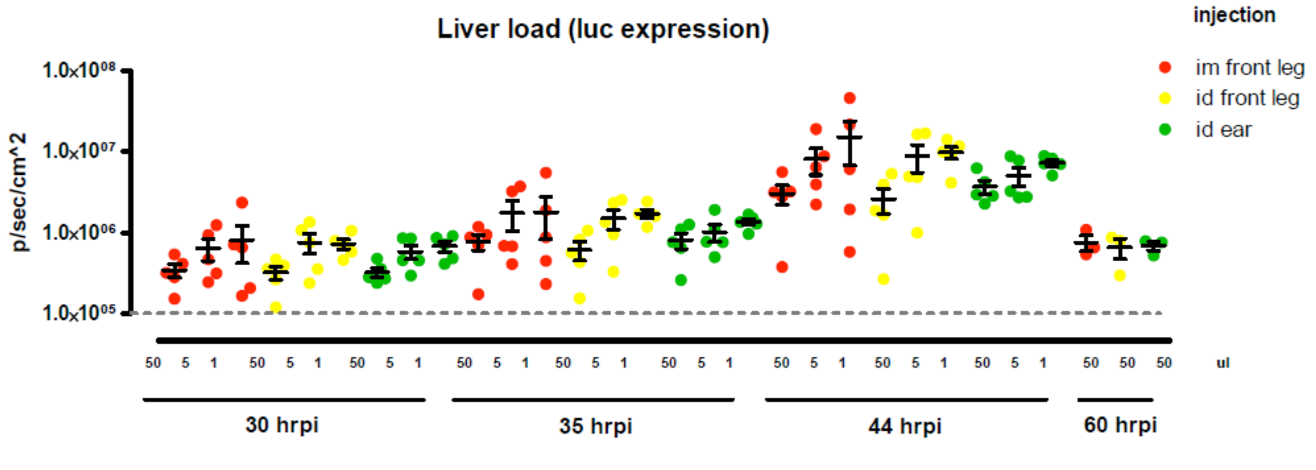

Figure 6: Liver Load after intramuscular and intradermal injection; Varying volume of administration. *Real time measurement of the liver load in mice injected with 5 x 10^4 sporozoites in different injection volumes by intramuscular or intradermal injection (n=5 per group) at different time points post injection (30, 35, 44 and 60 hours post infection). Dots represent the luminescent intensity (photon/sec/cm^2) of the regions of interest overlaying the livers from the individual mice. Dotted line represents the threshold of luminescent signalling.*[36]

Given the practical limitations of an intravenous challenge model and promising results already seen with intradermal administration of PfSPZ Challenge, it was decided at the EMVDA meeting that clinical testing of PfSPZ Challenge administered intramuscularly, at multiple sites in small volumes should be a research priority.

Other experience using aseptic, purified, cryopreserved *P. falciparum* sporozoites

PfSPZ Vaccine is a candidate malaria vaccine manufactured by Sanaria.[37] It is manufactured using the same process as PfSPZ Challenge except that the mosquitoes carrying the PfSPZ Vaccine are irradiated to attenuate the sporozoites,

The safety and tolerability of the PfSPZ Vaccine in humans was assessed in a Phase 1 clinical trial in the United States in malaria-naïve healthy volunteers following multiple-dose subcutaneous (SC) or intradermal (ID) administration (Clinical trials.gov:NCT01001650). In this dose escalation study volunteers receive either;

1. Four doses of 7,500 PfSPZ/immunization (Group 1) (n= 14 planned, and n= 12 received all doses)
2. Four doses of 30,000 PfSPZ/immunization (Group 2) (n=22 planned , and n= 17 received all doses);
3. Four doses of 135,000 PfSPZ/immunization (Group 3) (n=22 planned , and n= 19 received all doses); or
4. Six doses of 135,000 PfSPZ/immunization (Group 4) (n=22 planned , and n= 17 received all doses).

Equal numbers of volunteers were immunized intradermally (ID) in the forearm or subcutaneously (SC) in the upper arm by needle and syringe. The trial was an open-label, dose-escalation study in 80 malaria-naïve, healthy adults aged 18-50 years conducted at the Naval Medical Research Center (NMRC) and University of Maryland Center for Vaccine Development (UMD-CVD).

The vaccine was safe and well tolerated. Approximately half of volunteers in each group had a local or general adverse event (AE) considered possibly, probably or definitely related (= related) to administration of vaccine. The 42 volunteers with related AEs experienced 38, 21, 49, and 52 AEs in Groups 1-4 respectively. Ninety-one percent of related AEs were Grade 1. Eight volunteers experienced 13 related Grade 2 AEs. These included headache, malaise, myalgia, nausea, fever, chills, arthralgia, fatigue, and musculoskeletal pain. One volunteer had one Grade 3 AE, fatigue, considered possibly, but unlikely related. There were no related serious (Grade 4) AEs. Increasing dose did not increase numbers of volunteers with an AE, and incidence of AEs decreased as volunteers received additional doses of vaccine. Thirty-four (34) volunteers (42.5%) had a laboratory abnormality during the immunization phase of the trial. Five volunteers experienced a Grade 2 laboratory abnormality. No volunteers experienced Grade 3 or 4 laboratory abnormalities following administration of vaccine.

4. STUDY OVERVIEW

This is an open label, human pilot study to optimise CHMI administered by PfSPZ Challenge. This follows promising data from a clinical trial of intradermally administered PfSPZ Challenge at RUNMC (Roestenberg et al. *manuscript in preparation).*

Volunteers will be inoculated with PfSPZ Challenge. The route of administration and dose will vary in order to identify the optimal regimen that achieves the greatest infection rate in volunteers with *Plasmodium falciparum*. All volunteers recruited will be healthy adults aged between 18 and 45 years. Safety and infectivity data will be collected for each of the regimens (Table 4).

STUDY GROUPS

Group Number	No. of volunteers	Total Dose of PfSPZ	Total Volume Administered	Route of Administration	Number of injection sites
1	6	2,500 sporozoites	50µl x 2*	Intradermal	Two*
2	6	2,500 sporozoites	50µl x 2*	Intramuscular	Two*
3	6	25,000 sporozoites	50µl x 2*	Intramuscular	Two*

Table 4: *Overview of trial groups. * 1 injection per deltoid area. Each injection is dosed in a separate syringe.*

Initially volunteers will be able to opt into any group. Once one group has the required number of volunteers then subsequent volunteers will be allocated to the other groups. Volunteers and clinical investigators will not be blinded to group allocation, however laboratory investigators processing blood films and samples for PCR analysis will be blinded to group allocation.

In addition to the 18 volunteers to be enrolled in the study, back-up volunteers who have been screened and are eligible to participate in the study will be identified. These volunteers will be available to be enrolled in the study at short notice should a planned volunteer withdraw consent or become in-eligible immediately prior to challenge. Backup volunteers will be asked to be available to be enrolled in the study at short notice and may be asked to attend clinic on enrolment days in case another volunteer is found to be ineligible on enrolment day.

OBJECTIVES

Primary Objective
To determine the infectivity rates of PfSPZ Challenge administered in various regimens.

Secondary Objective
To assess the safety of PfSPZ Challenge administered in various regimens.

Tertiary Objectives
To determine the dynamics of *Plasmodium falciparum* parasite growth following PfSPZ Challenge administered in various regimens.

RATIONALE FOR TRIAL DESIGN

The choice of dose and route of administration of PfSPZ Challenge in this study is based on experience using the PfSPZ Challenge in a CHMI trial conducted by RUNMC (see above and

investigator brochure for PfSPZ Challenge). Group 1 is the same dosing schedule as the low dose group in the RUNMC PfSPZ challenge. We have chosen the intramuscular route of administration for PfSPZ Challenge in groups 2 and 3 based on the favourable infectivity rate of this route of administration in murine studies. Since the optimal inoculating dose of PfSPZ Challenge administered intramuscularly is not known, two doses of PfSPZ Challenge administered intramuscularly will be evaluated; 2,500 (group 2) and 25,000 (Group 3) sporozoites. The highest dose of sporozoites to be administered in this study (25,000 sporozoites) has already been safely administered to healthy volunteers in RUNMC by the intradermal route (see above). This trial will be the first time aseptic, purified, cryopreserved *P. falciparum* sporozoites (PfSPZ Challenge or PfSPZ Vaccine) have been administered intramuscularly to humans.

ADMINISTRATION OF PfSPZ CHALLENGE

The 6 volunteers in Group 1 and 2 volunteers from group 2 will be enrolled first on the same day. In the absence of any safety concerns in these volunteers, at least 48 hrs later the remaining 4 volunteers in Group 2 and 2 volunteers from Group 3 will be enrolled. In the absence of any safety concerns in these volunteers, at least 48 hrs later the remaining volunteers in Group 3 will be enrolled.

DURATION OF STUDY

The duration of involvement in the study from enrolment will be approximately 3 months.

DEFINITION OF START AND END OF TRIAL

The start of the trial is defined as the date of the first inoculation of the first volunteer. The end of the trial is the date of the last visit of the last volunteer.

POTENTIAL RISKS FOR VOLUNTEERS

Phlebotomy:

The maximum volume of blood drawn over the study period (556 mls over 3 months) should not compromise these otherwise healthy volunteers. There may be minor bruising, local tenderness or pre-syncopal symptoms associated with venepuncture, which will not be documented as AEs if they occur.

Administration of PfSPZ Challenge:

Serious allergic reactions including anaphylaxis have not been seen in the small number of individuals that have been inoculated with PfSPZ Challenge to date, but may occur and for this reason volunteers will be inoculated in a clinical area where Advanced Life Support trained physicians, equipment and drugs are immediately available for the management of any serious adverse reactions.

Plasmodium falciparum Infection

Volunteers are likely to develop symptomatic malaria infection following inoculation of PfSPZ Challenge. Symptoms and signs will include feverishness, fever, tachycardia, hypotension, chills, rigors, sweats, headache, anorexia, nausea, vomiting, diarrhoea, myalgia, arthralgia, low back pain, thrombocytopenia and lymphopenia. 30-50% of volunteers are likely to experience at least one severe adverse event related to *P. falciparum infection* (Roestenberg et al. *manuscript in preparation)* (section 7). Unmonitored and untreated, *P. falciparum* infection can be fatal and for this reason, volunteers will be followed up very closely post challenge and only enrolled in the study if they are deemed reliable and capable of complying with the intensive follow-up schedule (section 7). If necessary, volunteers may be admitted for in-patient care (section 7).

Treatment of *Plasmodium falciparum* Infection

Volunteers will be treated with oral Malarone unless there is a contraindication to this medication. Malarone is generally well tolerated but is associated with some side effects. Very common side effects, affecting more than 10% of people include headache, nausea and vomiting, stomach pain and diarrhoea. Common side effects that may affect up to 10% of people include dizziness, insomnia, strange dreams, depression, loss of appetite, fever and rash, which may be itchy and cough. Uncommon side effects that may affect up to 1% of people include anxiety, palpitations, swelling and redness of the mouth and hair loss.

Severe allergic severe reactions have occurred in a small number of people, but their exact frequency is unknown. Signs of severe allergic reactions include rash and itching, sudden wheezing, tightness of the chest or throat, or difficulty breathing, swollen eyelids, face, lips, tongue or other part of the body (See SmPC for side effects of and contraindications to Malarone).

Volunteers who have a contraindication to or are unable to tolerate Malarone will be treated with chloroquine or Riamet (see SmPC for side effects of and contraindications to chloroquine and Riamet).

POTENTIAL BENEFITS FOR VOLUNTEERS

Volunteers will not benefit directly from participation in this study. However, it is hoped that the information gained from this study will contribute to the development of a safe and effective malaria vaccine regimen. The only benefits for participants would be information about their general health status.

5. INVESTIGATIONAL PRODUCT – PFSPZ CHALLENGE

PfSPZ Challenge Product

PfSPZ Challenge are aseptic, cryopreserved *P. falciparum* sporozoites used for CHMI trials, produced by the biotechnology company; Sanaria (USA).[38] In brief, manufacture includes the production, under traditional environmental conditions, of eggs from a colony of *A. stephensi* mosquitoes housed in a controlled environmental chamber. Surface disinfection of the eggs is performed by exposure to chemical agents in a Class II biosafety cabinet (BSC). From this point forward, all materials and product are handled using aseptic methods to ensure that contaminating microorganisms are not introduced to and carried through the process. Surface-disinfected eggs are inoculated into sterile, vented flasks containing aseptic growth medium. The eggs hatch and develop into pupae, which are transferred to an adult mosquito container where the adult mosquitoes emerge. These adult mosquitoes, which have been raised under aseptic conditions, are fed *P falciparum* gametocyte-infected blood in a BSC in a High-Security Insectary in Rockville, Maryland, USA. The *P. falciparum* gametocyte-infected blood has been produced from cultures of the *P. falciparum* strain NF54 derived from a Master Cell Bank of the well-characterized *P. falciparum* strain NF54. Infected adult mosquitoes are maintained under aseptic conditions until *P. falciparum* sporozoites migrate to the salivary glands. The salivary glands from the *P. falciparum* sporozoite infected mosquitoes are removed by hand dissection. Salivary glands are then triturated to release the *P. falciparum* sporozoites. The sporozoites are purified, counted, and, at a specified concentration, cryopreserved. Cryopreservation commences with the addition of cryoprotective additives to the purified sporozoites to produce the PfSPZ Challenge product. PfSPZ Challenge is dispensed into screw-cap vials containing 15,000 or 50,000 sporozoites in a 20 µL aliquot. PfSPZ Challenge is stored in liquid nitrogen vapour phase at -140°C to -196°C.

PBS and HSA Diluent

The diluent for PfSPZ Challenge is composed of phosphate buffered saline (PBS) and human serum albumin (HSA). Vials of PBS and HSA will be shipped to the clinical site, where diluent composed of PBS and HSA is prepared according to SOP MC014 Storage, Preparation and Administration of PfSPZ Challenge Agent.

PBS that is manufactured in compliance with GMP and according to upstream processing specifications is purchased by Sanaria. Every lot of PBS is supplied with a Certificate of Analysis that is reviewed and approved upon receipt at Sanaria. The PBS is stored at ambient temperature in a controlled room.

Human serum albumin (HSA) (25%), which is approved for parenteral, intravenous administration to humans is purchased by Sanaria. Every lot of HSA is supplied with a Certificate of Analysis that is reviewed and approved upon receipt at Sanaria. HSA vials are stored at ambient temperature in a controlled room.

Storage & Handling of PfSPZ Challenge

PfSPZ Challenge is stored in liquid nitrogen vapour phase at -140°C to -196°C until it is shipped to a clinical study site. Shipment is in compliance with all U.S. Food and Drug Administration (FDA), U.S. Department of Transportation, and United Nations transport guidelines for shipping bio-hazardous materials on dry ice and liquid or vapour phase nitrogen.

Transfer of PfSPZ Challenge from its storage site to the clinical trial site will follow SOP MC014 Storage, Preparation and Administration of PfSPZ Challenge Agent. At the study site, the liquid nitrogen vapour phase container will be monitored. Receipt of the PfSPZ Challenge will be documented on a Tracking Log by study staff.

Preparation and Administration of PfSPZ Challenge

Each clinical site must confirm that the vials of PfSPZ Challenge have been transported and stored below -140°C. Immediately prior to use, PfSPZ Challenge in cryovials (each of which contains 15,000 or 50,000 sporozoites), will be thawed individually by partial submersion of the vials for 30 seconds in a 37°C ± 1°C water bath. Designated, trained study staff will then prepare, dilute (if necessary) and dispense PfSPZ Challenge to clinical staff at the clinical study site according to the appropriate SOP MC014 Storage, Preparation and Administration of PfSPZ Challenge Agent.

The diluent is PBS containing HSA. Aliquots of PBS and 25% HSA will be provided to the clinical sites by Sanaria Inc.

PfSPZ Challenge will be administered with a needle and syringe either intramuscularly or intradermally according to SOP MC014 Storage, Preparation and Administration of PfSPZ Challenge Agent. The study staff administrating PfSPZ Challenge will wear gloves and eye protection. During administration of PfSPZ Challenge, Advanced Life Support drugs and resuscitation equipment will be immediately available for the management of anaphylaxis.

Minimising environmental contamination with PfSPZ Challenge

The inoculation site will be covered with a dressing after inoculation. This should absorb any PfSPZ Challenge that may leak out through the needle track. The dressing will be removed from the injection site after 1 hour (+/- 5 minutes) and will be disposed of according to the appropriate SOP MC014 Storage, Preparation and Administration of PfSPZ Challenge Agent.

6. RECRUITMENT AND WITHDRAWAL OF TRIAL VOLUNTEERS

Volunteers

Volunteers may be recruited by use of an advertisement +/- registration form formally approved by the ethics committee and distributed or posted in the following places:

- In public places with the agreement of the owner / proprietor
- In newspapers or other literature for circulation
- On radio via announcements
- On a website operated by our group or with the agreement of the owner or operator (including on-line recruitment through our web-site)
- By e-mail distribution to a group or list only with the express agreement of the network administrator or with equivalent authorisation.
- By email distribution to individuals who have already expressed an interest in taking part in any clinical trial at the Oxford Vaccine Centre.
- On stalls or stands at exhibitions or fairs
- Via presentations (e.g. presentations at lectures or invited seminars)
- Direct mail-out: This will involve obtaining names and addresses of adults via the most recent Electoral Roll. The contact details of individuals who have indicated that they do not wish to receive postal mail-shots would be removed prior to the investigators being given this information. The company providing this service is registered under the Data Protection Act 1998. Investigators would not be given dates of birth or ages of individuals but the list supplied would only contain names of those aged between 18-45 years (as per the inclusion criteria).
- Oxford Vaccine Centre databases: We will contact individuals from databases of groups within the CCVTM, (including the Oxford Vaccine Centre database) of previous trial participants who have expressed an interest in receiving information about all future studies for which they may be eligible.

Informed Consent

All volunteers will sign and date the informed consent form before any study specific procedures are performed. The information sheet will be made available to the volunteer at least 24 hours prior to the screening visit. At the screening visit, the volunteer will be fully informed of all aspects of the trial, the potential risks and their obligations. The following general principles will be emphasised:

- Participation in the study is entirely voluntary
- Refusal to participate involves no penalty or loss of medical benefits
- The volunteer may withdraw from the study at any time
- The volunteer is free to ask questions at any time to allow him or her to understand the purpose of the study and the procedures involved
- There is no direct benefit from participating
- The volunteer's GP will be contacted to corroborate their medical history and confirm that the volunteer is eligible to take part in the study. Volunteers will only be enrolled in the study if written or verbal information regarding the volunteer's medical history is obtained from the GP.
- The volunteer will be registered on the TOPS database (The Overvolunteering Prevention System).

The aims of the study and all tests to be carried out will be explained. The volunteer will be given the opportunity to ask about details of the trial, and will then have time to consider whether or not to participate. If they do decide to participate, volunteers will be asked to complete a questionnaire testing their understanding of the trial.[18] This helps to ensure that individuals understand the trial sufficiently to give informed consent. Provided the volunteer answers all questions in the questionnaire correctly, they will be asked to sign and date two copies of the consent form, one for them to take away and keep, and one to be stored in the CRF. These forms will also be signed and dated by the Investigator. Volunteers who fail to answer all questions correctly on their first attempt will be allowed to re-take the questionnaire following further discussion with the investigator. Provided they subsequently answer all questions in the quiz correctly they may then complete the consent form and be screened for the trial.

Inclusion Criteria

The volunteer must satisfy all the following criteria to be eligible for the study:

- Healthy adults aged 18 to 45 years
- Able and willing (in the Investigator's opinion) to comply with all study requirements
- Willing to allow the investigators to discuss the volunteer's medical history with their General Practitioner
- Women only: Must practice continuous effective contraception for the duration of the study.
- Agreement to refrain from blood donation during the course of the study and for at least 3 years after the end of their involvement in the study.
- Written informed consent to undergo CHMI.
- Reachable (24/7) by mobile phone during the whole study period.
- Willingness to take a curative anti-malaria regimen.
- For volunteers not living in Oxford: agreement to stay in a hotel room close to the trial centre during a part of the study (At least Day 6.5 post inoculation until 2 days after treatment commenced).
- Answer all questions on the informed consent quiz correctly.

Exclusion Criteria

The volunteer may not enter the study if any of the following apply:

- History of clinical *P. falciparum* malaria.
- Travel to a malaria endemic region during the study period or within the preceding six months with positive *P. falciparum* serology at screening.
- Use of systemic antibiotics with known antimalarial activity within 30 days of study enrolment (e.g. trimethoprim-sulfamethoxazole, doxycycline, tetracycline, clindamycin, erythromycin, fluoroquinolones and azithromycin)
- Receipt of an investigational product in the 30 days preceding enrolment, or planned receipt during the study period.
- Prior receipt of an investigational malaria vaccine.
- Any confirmed or suspected immunosuppressive or immunodeficient state, including HIV infection; asplenia; recurrent, severe infections and chronic (more than 14 days) immunosuppressant medication within the past 6 months (inhaled and topical steroids are allowed).
- Use of immunoglobulins or blood products within 3 months prior to enrolment.
- History of sickle cell anemia, sickle cell trait, thalassemia or thalassemia trait.

- Pregnancy, lactation or intention to become pregnant during the study
- A history of allergic disease or reactions likely to be exacerbated by malaria infection.
- Contraindications to the use of all three proposed anti-malarial medications; Malarone, Riamet and Chloroquine.
- History of cancer (except basal cell carcinoma of the skin and cervical carcinoma in situ).
- History of serious psychiatric condition that may affect participation in the study.
- Any other serious chronic illness requiring hospital specialist supervision.
- Suspected or known current alcohol abuse as defined by an alcohol intake of greater than 42 units every week.
- Suspected or known injecting drug abuse in the 5 years preceding enrolment.
- Seropositive for hepatitis B surface antigen (HBsAg).
- Seropositive for hepatitis C virus (antibodies to HCV).
- An estimated, ten year risk of fatal cardiovascular disease of ≥5%, as estimated by the Systematic Coronary Risk Evaluation (SCORE) system.[39]
- Positive family history in 1st and 2nd degree relatives < 50 years old for cardiac disease.
- Volunteers unable to be closely followed for social, geographic or psychological reasons.
- Any clinically significant abnormal finding on biochemistry or haematology blood tests, urinalysis or clinical examination.
- Any other significant disease, disorder or finding which may significantly increase the risk to the volunteer because of participation in the study, affect the ability of the volunteer to participate in the study or impair interpretation of the study data.

Exclusion Criterion on Day of Challenge

- Acute disease, defined as moderate or severe illness with or without fever.
- Pregnancy

Withdrawal of Volunteers

In accordance with the principles of the current revision of the Declaration of Helsinki (updated 2008) and any other applicable regulations, a volunteer has the right to withdraw from the study at any time and for any reason, and is not obliged to give his or her reasons for doing so. The Investigator may withdraw the volunteer at any time in the interests of the volunteer's health and well-being. In addition the volunteer may withdraw/be withdrawn for any of the following reasons:

- Administrative decision by the Investigator
- Ineligibility (either arising during the study or retrospectively, having been overlooked at screening)
- Significant protocol deviation
- Volunteer non-compliance with study requirements
- An AE, which requires discontinuation of the study involvement or results in inability to continue to comply with study procedures.

The reason for withdrawal will be recorded in the CRF. If withdrawal is due to an AE, appropriate follow-up visits or medical care will be arranged, with the agreement of the volunteer, until the AE has resolved, stabilised or a non-trial related causality has been assigned. Any volunteer who is withdrawn from the study may be replaced, if that is possible within the specified time frame. The Local Safety Monitor (LSM) may recommend withdrawal of volunteers.

Any volunteer who fails to attend for two or more follow-up visits will be deemed to have withdrawn from the study. If a volunteer withdraws/is withdrawn from the study after receiving PfSPZ Challenge but before reaching the criterion for malaria diagnosis (Table 6), a complete, appropriate, curative course of anti-malarial therapy must be completed. The importance of this will be emphasised to volunteers at screening.

If a volunteer withdraws from the study, blood samples collected before their withdrawal from the trial will be used/stored unless the volunteer specifically requests otherwise. Data from volunteers withdrawn from the study before fulfilling the criterion for malaria diagnosis (Table 5) will be excluded from the analysis of results relating to the study's primary objective. Data from volunteers withdrawn from the study after fulfilling the criterion for malaria diagnosis will be included in analysis of results relating to the study's primary objective.

Pregnancy

Should a volunteer become pregnant during the trial, she will be followed up as other volunteers and in addition will be followed until pregnancy outcome. We will not routinely perform non-essential venepuncture on such volunteers. The management of any volunteers found to be pregnant at the point of diagnosis with malaria will be discussed with the on-call infectious diseases consultant at the Oxford Radcliffe Hospitals' Trust.

7. TREATMENT OF TRIAL VOLUNTEERS

STUDY PROCEDURES

Procedures will be performed at the time points indicated in the schedule of procedures (Table 5). Additional procedures or laboratory tests may be performed, at the discretion of the investigators if clinically necessary (e.g. urine microscopy in the event of positive urinalysis).

Observations

Pulse, blood pressure and temperature will be measured at the time points indicated in the schedule of procedures (Table 5).

Blood Tests

Blood will be drawn at the time points indicated in the schedule of procedures (Table 5) and the following laboratory assays performed;

1. At Oxford Radcliffe Hospitals NHS Trust Laboratory using NHS standard procedures:
   - **Haematology;** Full Blood Count
   - **Biochemistry;** Sodium, Potassium, Urea, Creatinine, Albumin, Liver Function Tests, Cholesterol
   - **Diagnostic serology;** HBsAg, HCV antibodies, HIV antibodies (Counselling will be given prior to testing blood for these blood-borne viruses)

2. At the Jenner Institute research laboratories:
   - **Diagnostic Tests:** Blood films for malaria parasites, PCR for parasite DNA, *P. falciparum* serology.
   - **Immunology;** Tests of immunogenicity of the PfSPZ will be performed at the discretion of the investigators and may include gene expression and human genetic studies.

Immunological assays will be conducted according to the procedures established in the test laboratories. With the volunteers' informed consent, any leftover cells and serum will be frozen for up to 15 years for future immunological analysis of malaria-specific responses (A Study of Exploratory Immunological Assays to Provide a Laboratory Based Correlate of Protection From Malaria; OXREC Number: 06/Q1606/123).

Urinalysis

Urine will be tested for the presence of clinically significant proteinuria, glucosuria or haematuria at screening and at various follow-up time points. For female volunteers only, urine will be tested for beta-human chorionic gonadotrophin (β HCG) at screening and the day before enrolment and immediately before anti-malarial therapy is started.

Administration of PfSPZ Challenge

PfSPZ Challenge will be administered as described above (section 5). The injection sites will be covered with a sterile dressing and the volunteer will stay in the clinical area for 1 hour (+/- 5 minutes) post challenge. The sterile dressing will be removed, injection sites inspected and physical observations performed at 1 hour post inoculation (+/- 5 mins). An oral thermometer will

be given to each volunteer along with the emergency 24 hour telephone number to contact the on call study physician if needed.

CLINICAL REVIEWS

All clinical reviews and procedures will be undertaken by one of the clinical team. The procedures to be included in each visit are documented in Table 4. Each review is assigned a time point and a window period within which the review will be conducted.

Screening visit

All potential volunteers will have a screening visit, which may take place up to 90 days prior to enrolment. Informed consent will be taken before screening as described above. If consent is obtained, the screening procedures indicated in the schedule of procedures (Table 5) will be undertaken. To avoid unnecessary additional venepuncture, if the appropriate blood test results for screening are available for the same volunteer from a screening visit for another Jenner Institute Clinical Trials group vaccine study, these results may be used for assessing eligibility (provided the results date within the 3 months preceding enrolment in VAC049).

Abnormal clinical findings from the medical history, physical examination or blood tests at any point in the study will be assessed using the table in Appendix A. If a test is deemed clinically significant it may be repeated to ensure it is not a single occurrence. If an abnormal finding is deemed to be clinically significant, the volunteer will be informed and appropriate medical care arranged with the permission of the volunteer. Decisions to exclude the volunteer from the enrolling in the trial or to withdraw a volunteer from the trial will be at the discretion of the Investigator following procedures for AEs as described in section 9.

Day before Challenge (C-1)

All volunteers will be seen the day before challenge. Assessment of any new medical issues or symptoms that have arisen since screening will be assessed. Physical observations (including measurement of height and weight), urine β HCG test in female volunteers and venepuncture for baseline exploratory immunology, PCR and safety bloods will be undertaken. The inclusion and exclusion criterion for the study will be reviewed. Results of safety bloods taken at this visit must be available and reviewed prior to challenge.

Day of Challenge (C0)

All volunteers will have physical observations performed prior to challenge. PfSPZ Challenge will be administered as described above (section 5). A Medic-Alert type card will be issued to each volunteer with information including Malarone/Riamet®/chloroquine/ sensitivity of the challenge malaria strain, study physician contact details and a request that the research team be contacted immediately in the event of illness/accident. Each subject will also be issued with an accurate oral thermometer. If the subject does not have their own mobile telephone they will be issued with one for the duration of the study, and counselled about the importance of keeping it switched on or checking the messages regularly. In addition full contact details for each subject will be documented, including home address, home and work land-line telephone numbers where available, and next-of-kin address and telephone numbers. Subjects must also provide the investigators with the name and 24 hour telephone number of a close friend, relative or housemate who lives nearby and will be kept informed of their whereabouts for the duration of the study.

Day After Challenge (C1)

All volunteers will be reviewed in clinic on the day following challenge. Physical observations will be performed and reactogenicity of PfSPZ Challenge assessed.

Days 2-6 Post Challenge (C2 - C5)

The liver stage of malaria infection is asymptomatic lasting approximately one week. For this reason volunteers will not be reviewed in clinic on Days C2-5 post challenge. However, they will be phoned daily by the clinic team during this period to make sure they are well and contactable, and will be able to contact the study physicians on the 24 hour emergency telephone number if needed.

Days 6.5 – 23 Post Challenge (C6.5 – C23)

From the evening of Day 6 until the evening of Day 14 post challenge, all volunteers will be reviewed twice daily. From Day 15 until Day 21 post challenge, volunteers will be reviewed daily.

Follow-up visits of all volunteers in this intensive post challenge phase will take place at the Centre for Clinical Vaccinology & Tropical Medicine (CCVTM) in Oxford. All volunteers will be seen by one of the clinical study team at each visit. The investigators are all physicians with extensive experience in acute medicine and infectious diseases and familiarity assessing patients with malaria. The Oxford based research nurses are qualified RNs with previous experience conducting CHMI trials.

At each follow-up visit:

- Physical observations will be checked.
- Venepuncture will be performed as per schedule of attendance (Table 5).
- Volunteers will be questioned as to whether they have
  - Experienced any of the foreseeable symptoms of malaria (Section 9)
  - Experienced any other symptoms
  - Taken any medications including over the counter medications

Subjects will be encouraged to contact one of the investigators on the 24 hour emergency mobile telephone number if they develop symptoms of malaria or concerning AEs between the regular reviews. The investigator will consider an extra clinical review and thick film microscopy if the subject's symptoms are moderate or severe. The severity of symptoms will be assessed using grading criterion summarised below. If a volunteer is unwell and unable to attend the CCVTM for a visit, they will be visited at home by one of the Investigators. Such visits will be conducted according to SOP VC020 Procedures for Lone Working on Jenner Institute Clinical Trials.

Timeline (days in relation to challenge)	Screening	C-1	C	C1	C6.5	C7	C7.5-C14	C15-C23	C35	C90
**Attendance Number**	1	2	3	4	5	6	7-20	21-29	30	31
**Window (days)**	N/A	-1	0	0	0	0	0	0	±5	±14
**Medical History**	X	(x)	(x)	(x)	(x)	(x)	(x)	(x)	(x)	(x)
**Clinical Assessment**	X	(x)	(x)	(x)	X	(x)	(x)	(x)	(x)	(x)
**Urinalysis**	X									
**Urinary β H-CG**[£]	X	X			(x)	(x)	(x)	(x)		
**Physical Observations**	X	X	X	X	X	X	X	X	X	X
**Informed Consent Quiz**	X									
**Medic Alert Card & Thermometer given to Volunteers**			X							
**Local & systemic events**				X	X	X	X	X	X	X
**Treatment for Malaria**					(x)	(x)	(x)	X		
**Thick Smear / PCR for malaria**		3			3	3	3 x 14	3 x 9		
**HBV, HCV, HIV (mL)**	5									
**Haematology (mL)**	2	2					2^		2	2
**Biochemistry (mL)***	3	3					3^		3	3
**Anti- *P. Falciparum* serology (mL)**[$]	2									
**Exploratory Immunology**	0	70		3		70**	90**	70**	70	70
**Blood volume per visit (mL)**	12	81	0	3	3	73	137	97	75	75
**Cumulative blood volume (mL)**	12	93	93	96	99	172	309	406	481	556

Table 5: *Schedule of clinical reviews*

C-1= Day Before Challenge, C6.5=6.5 days after challenge. (x)=If necessary. (Windows refer to time since last visit)

** Biochemistry will include Sodium, Potassium, Urea, Creatinine, Albumin, Liver Function Tests and Cholesterol. Cholesterol will only be tested at screening*

***70mls blood will be drawn for explorative immunology on C7, C11 and C21 if persistently undiagnosed with malaria. 10mls of blood will be drawn for cytokine analysis on C9 and C14 if persistently undiagnosed with malaria. If diagnosed with malaria, 70mls of blood will be drawn within 24 hours of diagnosis (but not if diagnosed on C7, C7.5, C11 or C11.5) and then no further blood drawn for explorative immunology until C35.*

^Blood will be drawn for haematology & biochemistry on C9 and within 24 hours of diagnosis. If a volunteer is slide positive before C9 then blood will be drawn for biochemistry and haematology within 24 hours of diagnosis and then not again until C35.

[£]*Urinary β HCG will be performed in female volunteers prior to commencing anti-malaria treatment*

[$]*Anti-P.Falciparum serology may be tested at screening in certain individuals to help determine prior malaria exposure*

Malaria Diagnosis

Diagnosis of malaria infection following challenge will be defined as positive thick film microscopy (at least one morphologically normal malaria trophozoite seen in 200 high-power (1000x) fields) by one or more experienced microscopists in a patient with symptoms suggestive of malaria.

Real time quantitative PCR for *P. falciparum* will simultaneously be performed, either once or twice a day, although Investigators (except the Chief Investigator) will be blinded to the results. If a positive thick film for malaria parasites is seen in an asymptomatic volunteer, the most recent PCR results for this volunteer will be un-blinded and the volunteer treated only if any available PCR result for that individual has been measured as >500 parasites/ml. In this scenario, if all available PCR results are <500 parasites/ml, treatment will be delayed until either the patient develops a further positive thick film in the presence of symptoms suggestive of malaria infection in the opinion of the Investigator, or the volunteer has a further positive thick film with a PCR measurement above 500 parasites/ml (Table 6).

Should a volunteer describe symptoms or display signs which are highly likely to represent malaria infection in the opinion of Investigators (such as fever, rigors or severe symptomatology) despite having a negative thick film and the absence of an alternative cause, clinicians may be un-blinded to the PCR result. If this is positive, the volunteer will be treated for malaria. As the PCR technique is still under development and not a validated assay, it cannot be used as the sole primary diagnostic endpoint in this study, but will be used by the Chief Investigator to provide additional safety and diagnostic information in the assessment of volunteers post-challenge.

	**THICK FILM MICROSCOPY**	
**MALARIAL SYMPTOMS**	**Positive**	**Negative**
**Symptomatic**	Successfully Infected	Successfully infected if any available PCR result is > 500 parasites/ml
**Asymptomatic**	Successfully infected if any available PCR result is > 500 parasites/ml<br>(Otherwise delay treatment)	Not infected

Table 6: *Malarial Diagnosis for Primary Endpoint Evaluation*

The investigators are able to treat any volunteer for malaria regardless of the thick film microscopy or PCR result if they are clinically concerned (and have discussed the case with the Chief Investigator), or a volunteer wishes to withdraw from the study.

When a case of malaria is diagnosed, each subject will have a clinical evaluation by one of the investigators (a physician) with appropriate history and physical examination where deemed to be necessary. If necessary, they can be admitted to the John Warin infectious Diseases ward at the Churchill Hospital, Oxford for observation and further medical management under the care of the Infectious Diseases Consultant on call.

Malaria Management

Volunteers will be treated with oral Malarone on diagnosis (see SmPC for Malarone). Malarone is a licensed drug in the UK for treatment of acute uncomplicated malaria caused by *Plasmodium falciparum*. Malarone is a combination drug consisting of proguanil hydrochloride and atovaquone. A treatment course of Malarone consists of 4 ‘standard tablets’ of Malarone (proguanil hydrochloride 100mg, atovaquone 250mg) once daily, orally for 3 days. All three doses of

Malarone will be directly observed in clinic. The infecting parasites are known to be fully sensitive to Malarone and this was the drug used in the CHMI PfSPZ Challenge trial undertaken at RUNMC.

Prior to starting Malarone, volunteers will be screened for drug interactions and contraindications (including a urinary β HCG test in female volunteers). Volunteers will be reminded of the potential side effects of Malarone and given the patient information sheet for Malarone.

Volunteers who remain undiagnosed with malaria at Day 21 (Table 6) will start a treatment course of Malarone at their day 21 visit.

If a patient is unable to tolerate an oral anti-malarial, the volunteer will be admitted for inpatient care and an appropriate parenteral anti-malarial therapy prescribed following discussion with the on call infectious diseases consultant covering the John Warin Ward (Infectious Diseases Unit, Oxford Radcliffe Hospitals' Trust).

If a volunteer withdraws/is withdrawn from the study after receiving PfSPZ Challenge but before reaching the criterion for malaria diagnosis (Table 6), a complete, appropriate, curative course of anti-malarial therapy must be completed. The importance of this will be emphasised to volunteers at screening.

Malaria Management – Alternative Anti-Malarial Medications

If a volunteer has a contraindication to Malarone or is unable to tolerate Malatone, oral chloroquine or Riamet (artemether & lumefantrine) may be prescribed as an alternative treatment for malaria.

Riamet (See SmPC for Riamet) is a licensed drug in the UK for treatment of acute, uncomplicated malaria caused by *Plasmodium falciparum*. Riamet is a combination drug consisting of 20mg artemether and 120mg lumefantrine per tablet. The NF54 parasites are known to be fully sensitive to Riamet. Prior to starting Riamet volunteers will be screened for drug interactions and contraindications to Riamet (including a urinary β HCG test in female volunteers). Volunteers will be advised to avoid grapefruit juice. Volunteers will be reminded of the potential side effects of Riamet, given the patient information sheet for Riamet and a card outlining when their doses of Riamet should be taken. A treatment course of Riamet consists of 6 doses of 4 tablets. The first 4 tablets will be given when diagnosis is made, followed by additional doses after 8, 24, 36, 48 and 60 hours (window period +/- 1 hour for each dose). Tablets should be taken together with a meal (a light snack will provided when doses are observed in clinic). Treatment will be observed on at least 3 occasions.

Chloroquine (See SmPc for chloroquine) is a licensed drug in the UK for treatment of acute, uncomplicated malaria caused by *Plasmodium falciparum*. The NF54 parasites are known to be fully sensitive to chloroquine. Prior to starting chloroquine, volunteers will be screened for drug interactions and contraindications to chloroquine. A urinary β HCG test will be performed in female volunteers. Volunteers will be reminded of the potential side effects of chloroquine, given the patient information sheet for chloroquine and a card outlining when their doses of chloroquine should be taken. A treatment course of chloroquine consists of tablets containing 155mg of chloroquine base administered as 620mg (4 tablets) orally at time 0, then 310mg (2 tablets) at 8 hours, 310mg (2 tablets) at 24 hours and 310mg (2 tablets) at 48 hours (window period +/- 1 hour for each dose). The treatment administration will be directly observed by one of the investigators or the research nurse, for the doses at 0, 24 and 48 hours.

Malaria Management – Supportive Medications

On development of symptoms, provided there are no contraindications, all volunteers will be provided with a 3 day course of paracetamol (1g orally up to four times a day) and a 3 day course of cyclizine (50mg orally three times a day) (See SmPC for Paracetamol & Cyclizine). Volunteers will be given the patient information sheet for these medications and advised how frequently they can take doses. Volunteers will be issued with a medication diary card on which they will be asked to document all doses of medications taken post challenge.

All medications used in trial will be handled and dispensed according to SOP VC021: Handling, Storage and Dispensing of Non-IMP Medication.

Criteria for Hospital Admission

If any of the following criteria are met, admission to the John Warin Ward (Infectious Diseases Unit, Oxford Radcliffe Hospitals' Trust) will be considered:

- Failure of symptoms to improve within 48 hours of starting anti-malarial therapy
- Unable to tolerate oral Malarone or alternative
- Dehydration requiring intravenous fluid therapy
- Signs or symptoms suggestive of pulmonary oedema
- Signs or symptoms of neurological dysfunction including altered consciousness
- Signs, symptoms or laboratory evidence of significant renal dysfunction
- Unanticipated concern about subject's home circumstances
- Any other significant finding which the Investigator feels warrant inpatient admission.

Ultimately, the decision regarding admission will be taken by the investigators in conjunction with the Infectious Diseases Consultant on call.

Follow-up Post Diagnosis

Subjects will be reviewed in clinic approximately 24 and 48 hours after diagnosis (and the start of anti-malarial therapy), when physical observations, symptom questionnaire and venepuncture for PCR and blood film will be performed (Table 5). If blood films taken at 24 and 48 hour post diagnosis are negative for parasites and the patient is asymptomatic or has mild, resolving symptoms, the volunteer will not be seen again in clinic until Day 35 post challenge (C35). If not, the volunteer will continue to be reviewed in clinic daily until they have 2 consecutive negative blood films at least 24 hours apart following start of antimalarial treatment, and all symptoms are mild or resolving. Volunteers will be given a card on which to document the end of date of any outstanding malaria symptoms on-going between completing anti-malarial therapy and Day 35 post challenge.

Safety Measures for Challenge

Volunteer safety is of paramount importance. The following measures are in place to safeguard volunteer safety;

- Volunteers will only be enrolled in the study if both investigators and the volunteers' GP's feel this is appropriate.
- Volunteers' understanding of the trial information will be tested by means of a questionnaire at screening. This provides further confidence that fully informed consent has been obtained.
- If the subject does not have their own mobile telephone they will be issued with one for the duration of the study and counselled about the importance of keeping it switched on or checking the messages regularly.
- Before challenge, full contact details for each subject will be documented, including home address and mobile telephone numbers. Mobile telephone numbers will be verified prior to challenge to ensure the volunteers are easily contactable. Home and work land-line telephone numbers where available and next-of-kin address and telephone numbers will also be documented. Subjects must also provide the investigators with the name and 24 hour telephone number of a close friend, relative or housemate who lives nearby and will be

kept informed of their whereabouts for the duration of the study.

- On the day of challenge volunteers will be provided with a medic alert card containing contact details for the study team, brief details of the study and the drug sensitivities of PfSPZ Challenge.
- On days 2 to 5 post challenge when the volunteers do not have scheduled clinic visits, volunteers will be phoned daily by the clinic team in order to ensure they are well and contactable.
- Volunteers will be able to contact a medically qualified member of the study team 24 hours a day throughout the study period and will be instructed to contact the investigator immediately should they manifest any signs or symptoms they perceive as serious.
- If necessary, the study team will visit volunteers in their own homes if they are unable to attend clinic for review.
- All doses of Malarone will be observed by the study team. (For volunteers taking other anti-malarials, at least half of all doses will be observed).
- Volunteers will be counselled that should they fail to return for treatment having been infected with *P. falciparum* they could become very unwell and potentially die. They will be instructed to remain in Oxford and the immediate surrounding area for the duration of the intensive follow-up schedule (Days 1-23 post challenge). They will be informed that should they fail to attend a scheduled clinic visit post challenge, their nominated contact, next of kin and the police may be informed and a search started.
- Volunteers will be counselled to contact the study team or their GP if they feel feverish or unwell in the 6 months following the challenge.

Measures to be taken if a Volunteer Goes Missing Post Challenge

In the unlikely event that a volunteer should (a) fail to attend for a scheduled clinical visit or (b) be un-contactable by telephone after being inoculated with PfSPZ Challenge and before completion of an appropriate course of anti-malaria therapy, the following stakeholders will be informed;

- All investigators
- The volunteer's nominated contact and next of kin
- The trial sponsor
- The IND sponsor
- The local safety committee
- The local REC
- The competent authority
- Relevant hospital trust R&D departments
- The local police department
- Local Accident and Emergency departments

All efforts will be made to locate the volunteer by the police. While all parties will aim to preserve the volunteer's confidentiality, if necessary, details of the volunteer's identity and participation in the study may be passed to the national media in order to help locate the missing individual. Volunteers will be informed of this during screening.

Day 35 Post Challenge (C35)

All volunteers will be reviewed in clinic 35 days following challenge. Physical observations will be performed and AEs assessed. Venepuncture will be performed (Table 5).

Day 90 Post Challenge (C90)

All volunteers will be reviewed in clinic 90 days following challenge. Physical observations will be performed and AEs assessed. Venepuncture will be performed (Table 5).

8. ASSESSMENT OF SCIENTIFIC OBJECTIVES

PRIMARY EVALUATION CRITERIA

The infectivity of the three administration regimens will be assessed by thick film microscopy and highly sensitive PCR for *Plasmodium falciparum* DNA.

Volunteers will be defined as being successfully infected at a time point if they fulfill the criteria in Table 6.

SECONDARY EVALUATION CRITERIA

The safety of PfSPZ Challenge administered by needle and syringe and the resultant *P. falciparum* infection will be assessed by analysing actively and passively collected data from clinical review of volunteers and laboratory measurements.

TERTIARY EVALUATION CRITERIA

The dynamics of *Plasmodium falciparum* parasite growth following administration of PfSPZ Challenge will be assessed by analyzing parasite multiplication rates using highly sensitive PCR for *Plasmodium falciparum* DNA.

9. ASSESSMENT OF SAFETY

Safety of PfSPZ Challenge will be assessed by analysing the frequency, incidence and nature of adverse events and serious adverse events arising during the study.

DEFINITIONS

The MHRA has confirmed that PfSPZ Challenge does not constitute an IMP. However, definitions for safety purposes are based on those used by the MHRA for IMPs.

Adverse Event (AE): An AE is any untoward medical occurrence in a volunteer, including a dosing error, which may occur during or after administration of PfSPZ Challenge and does not necessarily have to have a causal relationship with the intervention. An AE can therefore be any unfavourable and unintended sign (including an abnormal laboratory finding), symptom or disease temporally associated with the study intervention, whether or not considered related to the study intervention.

Adverse Reaction (AR): An AR is any untoward or unintended response to PfSPZ Challenge. This means that a causal relationship between PfSPZ Challenge and an AE is at least a reasonable possibility, i.e., the relationship cannot be ruled out. All cases judged by either the reporting medical investigator or the sponsors as having a reasonable suspected causal relationship to PfSPZ Challenge (i.e. possibly, probably or definitely related to PfSPZ Challenge) will qualify as adverse reactions.

Unexpected Adverse Reaction: An unexpected adverse reaction is where the nature or severity of the adverse reaction is inconsistent with that expected for the intervention.

Serious Adverse Event (SAE): To ensure no confusion or misunderstanding of the difference between the terms "serious" and "severe", which are not synonymous, the following note of clarification is provided: The term "severe" is often used to describe the intensity (severity) of a specific event (as in mild, moderate, or severe myocardial infarction); the event itself, however, may be of relatively minor medical significance (such as severe headache). This is not the same as "serious," which is based on patient/event outcome or action criteria usually associated with events that pose a threat to a volunteer's life or functioning. Seriousness (not severity) serves as a guide for defining regulatory reporting obligations.

An SAE is an AE that results in any of the following outcomes, whether or not considered related to the study intervention.

- Death (i.e., results in death from any cause at any time)
- Life-threatening event (i.e., the volunteer was, in the view of the investigator, at immediate risk of death from the event that occurred). This does not include an AE that, if it occurred in a more serious form, might have caused death.
- Persistent or significant disability or incapacity (i.e. substantial disruption of one's ability to carry out normal life functions).
- Hospitalisation, regardless of length of stay, even if it is a precautionary measure for continued observation. Hospitalisation (including inpatient or outpatient hospitalization for an elective procedure) for a pre-existing condition that has not worsened unexpectedly does not constitute a serious AE.
- An important medical event (that may not cause death, be life threatening, or require hospitalization) that may, based upon appropriate medical judgment, jeopardize the volunteer and/or require medical or surgical intervention to prevent one of the outcomes listed above. Examples of such medical events include allergic reaction requiring intensive

treatment in an emergency room or clinic, blood dyscrasias, or convulsions that do not result in inpatient hospitalization.

- Congenital anomaly or birth defect.

Serious Adverse Reaction (SAR): An adverse event (expected or unexpected) that is both serious and, in the opinion of the reporting investigator or sponsors, believed to be possibly, probably or definitely due to PfSPZ challenge or any other study treatments, based on the information provided.

Suspected Unexpected Serious Adverse Reactions (SUSARs): A SUSAR is a SAE that is unexpected and thought to be possibly, probably or definitely related to PfSPZ Challenge.

Foreseeable Adverse Drug Reactions:

PfSPZ Challenge is expected to cause *P. falciparum* clinical disease. The foreseeable AEs of clinical malaria include; fever, tachycardia, hypotension, feverishness, chills, rigor, sweats, headache, anorexia, nausea, vomiting, myalgia, arthralgia, low back pain, fatigue, lymphopenia and thrombocytopenia.

The foreseeable AEs following administration of the licensed medications; Malarone, Riamet, chloroquine, paracetamol and cyclizine are listed in the SmPCs for these medications.

Foreseeable Serious Adverse Events: Volunteers developing clinical *P. falciparum* disease may develop AEs that require in-patient admission and so would be deemed SAEs. All SAEs, foreseeable or not, will be notified to LSC as described below.

CAUSALITY ASSESSMENT

For each AE, an assessment of the relationship of the AE to the study intervention(s) will be undertaken. The relationship of the adverse event with the study procedures will be categorized as unrelated, unlikely to be related, possibly related, probably related or definitely related. An intervention-related AE refers to an AE for which there is a possible, probable or definite relationship to the study intervention. The investigator will use clinical judgment to determine the relationship. Alternative causes of the AE, such as the natural history of pre-existing medical conditions, concomitant therapy, other risk factors and the temporal relationship of the event to administration of PfSPZ Challenge will be considered and investigated.

Reporting Procedures for AEs (Excluding SAEs)

All AEs occurring during the study observed by the investigator or reported by the patient will be recorded in the CRF. AEs that result in a patient's withdrawal from the study or that are present at the end of the study will be followed up (if volunteer's consent to this) until a satisfactory resolution or stabilisation occurs, or until a non-study related causality is assigned.

The severity of clinical and laboratory adverse events will be assessed according to the scales in Tables 8-11.

Adverse Event	Grade	Intensity
Pain at injection site	1	Pain that is easily tolerated
	2	Pain that interferes with daily activity
	3	Pain that prevents daily activity
Erythema at injection site*	1	>3 - ≤50 mm
	2	>50 - ≤100 mm
	3	>100 mm
Swelling at injection site	1	>0 - ≤20 mm
	2	>20 - ≤50 mm
	3	>50 mm

Table 8: *Severity grading criterion for injection site pain, erythema and swelling. *erythema ≤3mm is an expected consequence of skin puncture and will therefore not be considered an adverse event*

**GRADE 0**	None
**GRADE 1**	Mild: Transient or mild discomfort (< 48 hours); no medical intervention/therapy required
**GRADE 2**	Moderate: Mild to moderate limitation in activity - some assistance may be needed; no or minimal medical intervention/therapy required
**GRADE 3**	Severe: Marked limitation in activity, some assistance usually required; medical intervention/therapy required, hospitalisation possible

Table 9: *Severity grading criterion for AEs.*

Physical Observations	Grade 1	Grade 2	Grade 3
**Tachycardia – beats per min**	101-115	116-130	>130
**Hypotension (diastolic) mm Hg**	85-89	80-84	<80
**Hypertension (systolic) mm Hg****	141-150	151-155	>155
**Hypertension (diastolic) mm Hg****	91-95	96-100	>100
**Fever °C**	37.6 – 38.0	>38.0	>39.0

Table 10: *Severity grading criterion for clinically significant abnormal physical observations. All observations should be measured at rest. *Only applies when resting heart rate is between 60 and 100 beats per minutes. Use clinical judgement when characterising bradycardia around some healthy subject populations (e.g. conditioned athletes). **Systolic or diastolic hypertension may only be confirmed as clinically significant (and therefore an AE) if persistently present when observations are repeated (i.e. isolated measurements of hypertension are not clinically significant).*[40]

Laboratory Test	Grade 1	Grade 2	Grade 3
Hgb (female) – decrease from testing laboratory LLN in gm/dl	>1.0 - <1.5	≥1.5 & <2.0	≥2.0
Hgb (male) – decrease from testing laboratory LLN in gm/dl	≥1.5 & <2.0	≥2.0 & <2.5	≥2.5
Absolute neutrophil count (ANC, cells/mm^3)	1000-1499	500-999	<500
Leukopenia (WBC, cells/mm^3)	<3500 - ≥2500	<2500 - ≥1500	<1500
Platelets (cells/mm^3)	125,000 – 135,000	100,000 – 124,000	20,000-99,000
Bilirubin – when accompanied by any increase in Liver Function Test increase by factor	1.1 – 1.25 x ULN	1.26 – 1.5 x ULN	1.51 – 1.75 x ULN
ALT	1.25 – 2.5 x ULN	>2.6 – 5.0 x ULN	>5.0 x ULN
Creatinine	1.1 – 1.5 x ULN	>1.6 – 3.0 x ULN	>3.0 x ULN

Table 11: *Severity grading criteria for clinically significant laboratory abnormalities*[40]

Reporting Procedures for Serious AEs

In order to comply with current regulations on serious adverse event reporting to Health Authorities, the event will be documented accurately and notification deadlines respected. All SAEs will be reported to an internal safety group by email within 1 working day of the investigators being aware of their occurrence, as described in SOP VC004 Safety reporting. Copies of reports will be forwarded to the IND sponsor. All SAE will be reported to the US FDA by the IND sponsor. SAEs will not normally be reported to the REC unless there is a clinically important increase in occurrence rate, an unexpected outcome, or a new event that is likely to affect safety of trial volunteers.

Reporting Procedures for SUSARS

The Chief Investigator will report all SUSARs to the REC within 15 days. The IND Sponsor will report all SUSARs to US FDA. The Chief Investigator will also inform all investigators concerned of relevant information about SUSARs that could adversely affect the safety of participants. In addition, the Chief investigator will report any SUSARs relating to licensed products used in the trial (Malarone, Riamet, Chloroquine, Paracetamol & Cyclizine) to the MHRA using the electronic 'Yellow Card' System.[41]

All deaths occurring during the study will be reported to the trial and IND Sponsor. For all deaths, available autopsy reports and relevant medical reports will be made available for reporting to the relevant authorities.

Procedures to be followed in the Event of Abnormal Findings

Abnormal clinical findings from medical history, examination or blood tests, will be assessed as to their clinical significance using the table in Appendix A. If a test is deemed clinically significant, it may be repeated, to ensure it is not a single occurrence. If a test remains clinically significant, the volunteer will be informed and appropriate medical care arranged as appropriate with the permission of the volunteer. Decisions to exclude the volunteer from the enrolling in the trial or to withdraw a volunteer from the trial will be at the discretion of the Investigator.

Local Safety Committee

A Local Safety Committee (LSC) will be appointed to provide real-time safety oversight. The LSC will review SAEs deemed possibly, probably or definitely related to study interventions. The LSC will be notified within 1 working day of the investigators' being aware of their occurrence. The LSC has the power to terminate the study if deemed necessary following a study intervention-related SAE. At the time of writing the LSC will be chaired by Dr Brian Angus, a Clinical Tutor in Medicine, Honorary Consultant Physician and Director, Centre for Tropical Medicine at the University of Oxford. There will be a minimum of two other appropriately qualified committee members as specified in SOP VC004 Safety reporting. All correspondence between investigator and LSC will be conveyed by the investigator to the trial Sponsor and IND Sponsor.

The chair of the LSC may be contacted for advice and independent review by the investigator, trial Sponsor or IND Sponsor in the following situations:

- Following any SAE deemed to be possibly, probably, or definitely related to a study intervention.
- Any other situation where the Investigator, trial sponsor or IND sponsor feels independent advice or review is important.

Safety hold of the study and stopping rules

The study may be placed on safety hold for the following reasons:

- On advice of the safety monitor

- On advice of the investigators
- On advice of the REC or Local Safety Committee
- One or more participants experience a SAE that is determined to be related to the study product administration.
- One or more grade 3 or higher adverse events which are unexpected and possibly or probably related to the challenge in any group of 6 subjects

If the study is placed on hold it may only be restarted following discussion with and approval from the local safety committee, the trial sponsor, IND sponsor and Chief investigator.

Safety Profile Review

The safety profile will be assessed on an on-going basis by the investigators. An internal safety group will also review safety issues and SAEs as they arise.

10. STATISTICS

This is an open label pilot study. The number of subjects in each group will be 6 and the total number of subjects enrolled will be 18.

In the great majority of human malaria challenge studies performed to date, group sizes have been in single figures. This reflects both practical and ethical limitations on the numbers of individuals that can be recruited and challenged safety. For this reason, this study is designed to assess proof of concept rather than to look for statistically significant associations and the sample size has been accordingly kept to the minimum.

Data analysis will consist primarily of descriptive summaries for treatment groups. The primary endpoint of successful malarial infection will be assessed for each patient according to the definition in (Table 5) and the number and percent of patients achieving this endpoint summarised for each treatment group.

For secondary and tertiary endpoints descriptive summaries and plots over the time course for both individual patient results and groups will be presented. Where appropriate highly skewed data will be log-transformed and presented as geometric means with 95% confidence intervals. Time to event data will be described using the Kaplan-Meier method.

11. QUALITY CONTROL AND QUALITY ASSURANCE PROCEDURES

Investigator procedures

Approved site-specific SOPs will be used at all clinical and laboratory sites.

Monitoring

Monitoring will be performed according to ICH Good Clinical Practice (GCP) by CTRG. Following written standard operating procedures, the monitors will verify that the clinical trial is conducted and data are generated, documented and reported in compliance with the protocol, GCP and the applicable regulatory requirements. The investigator sites will provide direct access to all trial related source data/documents and reports for the purpose of monitoring and auditing by the IND and trial sponsors and inspection by local and regulatory authorities.

Modification to protocol

No amendments to this protocol will be made without consultation with, and agreement of, the trial and IND Sponsors. Any amendments to the trial that appear necessary during the course of the trial must be discussed by the Investigator and trial and IND Sponsors concurrently. If agreement is reached concerning the need for an amendment, it will be produced in writing by the Chief Investigator and will be made a formal part of the protocol following ethical and regulatory approval.

An administrative change to the protocol is one that modifies administrative and logistical aspects of a protocol but does not affect the subjects' safety, the objectives of the trial and its progress. An administrative change does not require NHS REC or Competent authority approval. The Investigator is responsible for ensuring that changes to an approved trial, during the period for which NHS REC / Competent authority approval has already been given, are not initiated without NHS REC / Competent authority review and approval, except to eliminate apparent immediate hazards to the subject.

Protocol deviation

Any deviations from the protocol will be documented in a protocol deviation form and filed in the trial master file.

Audit & inspection

The QA manager at the trial site will conduct internal audits to check that the trial is being conducted, data recorded, analysed and accurately reported according to the protocol, trial SOPs and incompliance with ICH GCP. The audits will also include laboratory activities according to an agreed audit schedule taking into consideration the 2009 MHRA guidelines for GCP in the laboratory. The internal audits will supplement the external monitoring process and will review processes not covered by the external monitor.

The sponsor (both trial and IND) may carry out audit to ensure compliance with the protocol, GCP and appropriate regulations.

Trial Progress

The progress of the trial will be overseen by the Chief Investigator.

12. ETHICS

Declaration of Helsinki

The Investigator will ensure that this study is conducted according to the principles of the current revision of the Declaration of Helsinki 2008.

ICH Guidelines for Good Clinical Practice

The Investigator will ensure that this study is conducted in full conformity with relevant regulations and with the ICH guidelines for GCP (CPMP/ICH/135/95) July 1996.

Informed Consent

Written, informed consent will be obtained, as described above.

Research Ethics Committee (REC)

A copy of the protocol, proposed informed consent form, other written volunteer information and the proposed advertising material will be submitted to a REC for written approval. The Investigator will submit and, where necessary, obtain approval from the REC for all subsequent substantial amendments to the protocol and informed consent document.

Volunteer Confidentiality

All data will be anonymised: volunteer data will be identified by a unique study number in CRF and database. Separate confidential files containing identifiable information will be stored in secured locations in accordance with the Data Protection Act 1998. Only the sponsor (both trial and IND) representative, investigators, the clinical monitor, the REC and the MHRA will have access to the records. Photographs taken of PfSPZ Challenge administration sites (if required, with the volunteer's written, informed consent) will not include the volunteer's face and will be identified by the volunteer's identification number only. Once developed, photographs will be stored as confidential records, as above. This material may be shown to other professional staff, used for educational purposes, or included in a scientific publication.

13. DATA HANDLING AND RECORD KEEPING

Data Handling

The Chief Investigator will be the data manager with responsibility for delegating the receiving, entering, cleaning, querying, analysing and storing all data that accrues from the study. The investigators will enter the data into the volunteers' CRFs, which will be in a paper and/or electronic format (using an OpenClinica™ database stored on a secure University of Oxford server). This includes safety data, laboratory data (both clinical and immunological) and outcome data. Data is entered in a web browser on PCs and then transferred to the OpenClinica Database by encrypted (Https) transfer.

Record Keeping

The investigators will maintain appropriate medical and research records for this trial in compliance with ICH E6 GCP and regulatory and institutional requirements for the protection of confidentiality of volunteers. The Chief Investigator, co-investigators and clinical research nurses will have access to records. The investigators will permit authorized representatives of the sponsor(s) (both trial and IND sponsors), regulatory agencies and the monitors to examine (and when required by applicable law, to copy) clinical records for the purposes of quality assurance reviews, audits and evaluation of the study safety and progress.

Source Data and Case Report Forms (CRFs)

All protocol-required information will be collected in CRFs designed by the investigator. All source documents will be filed in the CRF. Source documents are original documents, data, and records from which the volunteer's CRF data are obtained. For this study these will include, but are not limited to; volunteer consent form, blood results, GP response letters, laboratory records and correspondence. In the majority of cases, CRF entries will be considered source data as the CRF is the site of the original recording (i.e. there is no other written or electronic record of data). In this study this will include, but is not limited to medical history, medication records, vital signs, physical examination records, urine assessments, blood results, adverse event data and details of study interventions. All source data and volunteer CRFs will be stored securely.

Data Protection

The study protocol, documentation, data and all other information generated will be held in strict confidence. No information concerning the study or the data will be released to any unauthorized third party, without prior written approval of the sponsor (both trial and IND).

14. FINANCING AND INSURANCE

Financing

The study will be funded primarily by support from the National Institute of Health Research (NIHR) Oxford Biomedical Research Centre (BRC) and potentially other funders.

Insurance

Negligent Harm: Indemnity and/or compensation for negligent harm arising specifically from an accidental injury for which the University is legally liable as the Research Sponsor will be covered by the University of Oxford.

Non-Negligent Harm: Indemnity and/or compensation for harm arising specifically from an accidental injury, and occurring as a consequence of the Research Subjects' participation in the trial for which the University is the Research Sponsor will be covered by the University of Oxford.

Compensation

Volunteers will be compensated for their time and for the inconvenience caused by procedures as below:

- Travel expenses	£6 per visit
- Inconvenience of blood tests:	£6 per blood donation
- Time required for visit:	£15 per hour
- Illness Compensation: 3 days	£360.

Backup volunteers (section 4) who are not enrolled in the study will be compensated £200. This is in addition to compensation for visits they may have attended.

15. APPENDIX A. LABORATORY VALUES FOR EXCLUSION

The following reference ranges are provided for the purpose of guidance only for Investigators during the trial. Results during the trial that fall out with these ranges may not be of clinical significance but should be considered on an individual basis. Abnormal results judged of clinical significance should ordinarily be recorded as adverse events (AEs).

PARAMETER	LOWER LIMIT OF EXCLUSION	UPPER LIMIT OF EXCLUSION
**BIOCHEMISTRY**		
Potassium [mmol/L]	<3.2	>5.5
Sodium [mmol/L]	<132	>148
Urea [mmol/L]	N/A	>9
Creatinine [μmol/L]	N/A	>145
Albumin [g/L]	<30	N/A
Total bilirubin [μmol/L]	N/A	>19 when accompanied by elevated liver enzyme
ALT [IU/L]	N/A	>56
ALP [IU/L]	N/A	>350
**HAEMATOLOGY**		
Haemoglobin [g/dL]	Male: < 11.5<br>Female: < 10.5	Male: > 18<br>Female: >17.5
White Cell Count [x 10^9/L]<br>Neutrophil count [x 10^9/L]	<3.5<br><1.5	>14.0
Platelet Count [x 10^9/L]	<136	>500
**URINE ANALYSIS (using MULTISTIX * 10 SG Bayer Diagnostics)**		
Protein	2+ or 0.5-1gm loss/day	
Blood [mmol/L]	2+ confirmed by 5-10 rbc/hpf	

16. REFERENCES

1. Okiro EA, Hay SI, Gikandi PW, et al. The decline in paediatric malaria admissions on the coast of Kenya. Malar J 2007;6:151.

2. WHO. World Malaria Report 2009. 2009.

3. Sachs J, Malaney P. The economic and social burden of malaria. Nature 2002;415:680-5.

4. White NJ. Antimalarial drug resistance. J Clin Invest 2004;113:1084-92.

5. Das P, Horton R. Malaria elimination: worthy, challenging, and just possible. Lancet 2010;376:1515-7.

6. Moxon R. Microbial Challenge Studies of Human Volunteers. The Academy of Medical Sciences 2005.

7. Sauerwein RW, Roestenberg M, Moorthy VS. Experimental human challenge infections can accelerate clinical malaria vaccine development. Nat Rev Immunol 2011;11:57-64.

8. Lees R. Treatment of General Paralysis of the Insane by Induced Malaria: Note on Fifty Cases. Br Med J 1931;2:336-9.

9. Powell RD, McNamara JV. Infection with chloroquine-resistant Plasmodium falciparum in man: prepatent periods, incubation periods, and relationships between parasitemia and the onset of fever in nonimmune persons. Ann N Y Acad Sci 1970;174:1027-41.

10. Trager W, Jensen JB. Human malaria parasites in continuous culture. Science 1976;193:673-5.

11. Ifediba T, Vanderberg JP. Complete in vitro maturation of Plasmodium falciparum gametocytes. Nature 1981;294:364-6.

12. Campbell CC, Collins WE, Nguyen-Dinh P, Barber A, Broderson JR. Plasmodium falciparum gametocytes from culture in vitro develop to sporozoites that are infectious to primates. Science 1982;217:1048-50.

13. Chulay JD, Schneider I, Cosgriff TM, et al. Malaria transmitted to humans by mosquitoes infected from cultured Plasmodium falciparum. Am J Trop Med Hyg 1986;35:66-8.

14. Herrington DA, Clyde DF, Losonsky G, et al. Safety and immunogenicity in man of a synthetic peptide malaria vaccine against Plasmodium falciparum sporozoites. Nature 1987;328:257-9.

15. Ballou WR, Hoffman SL, Sherwood JA, et al. Safety and efficacy of a recombinant DNA Plasmodium falciparum sporozoite vaccine. Lancet 1987;1:1277-81.

16. Church LW, Le TP, Bryan JP, et al. Clinical manifestations of Plasmodium falciparum malaria experimentally induced by mosquito challenge. J Infect Dis 1997;175:915-20.

17. Moorthy VS, Diggs C, Ferro S, et al. Report of a consultation on the optimization of clinical challenge trials for evaluation of candidate blood stage malaria vaccines, 18-19 March 2009, Bethesda, MD, USA. Vaccine 2009;27:5719-25.

18. Moorthy VS. Standardization of Design and Conduct of P. falciparum Sporozoite Challenge Trials. 2011.

19. Epstein JE, Rao S, Williams F, et al. Safety and clinical outcome of experimental challenge of human volunteers with Plasmodium falciparum-infected mosquitoes: an update. J Infect Dis 2007;196:145-54.

20. Verhage DF, Telgt DS, Bousema JT, et al. Clinical outcome of experimental human malaria induced by Plasmodium falciparum-infected mosquitoes. Neth J Med 2005;63:52-8.

21. Nieman AE, de Mast Q, Roestenberg M, et al. Cardiac complication after experimental human malaria infection: a case report. Malar J 2009;8:277.

22. Webster DP, Dunachie S, Vuola JM, et al. Enhanced T cell-mediated protection against malaria in human challenges by using the recombinant poxviruses FP9 and modified vaccinia virus Ankara. Proc Natl Acad Sci U S A 2005;102:4836-41.

23. Dunachie SJ, Walther M, Vuola JM, et al. A clinical trial of prime-boost immunisation with the candidate malaria vaccines RTS,S/AS02A and MVA-CS. Vaccine 2006;24:2850-9.

24. Dunachie SJ, Walther M, Epstein JE, et al. A DNA prime-modified vaccinia virus ankara boost vaccine encoding thrombospondin-related adhesion protein but not circumsporozoite protein partially protects healthy malaria-naive adults against Plasmodium falciparum sporozoite challenge. Infect Immun 2006;74:5933-42.

25. Walther M, Dunachie S, Keating S, et al. Safety, immunogenicity and efficacy of a pre-erythrocytic malaria candidate vaccine, ICC-1132 formulated in Seppic ISA 720. Vaccine 2005;23:857-64.

26. Walther M, Thompson FM, Dunachie S, et al. Safety, immunogenicity, and efficacy of prime-boost immunization with recombinant poxvirus FP9 and modified vaccinia virus Ankara encoding the full-length Plasmodium falciparum circumsporozoite protein. Infect Immun 2006;74:2706-16.

27. Porter DW, Thompson FM, Berthoud TK, et al. A human Phase I/IIa malaria challenge trial of a polyprotein malaria vaccine. Vaccine 2011.

28. Thompson FM, Porter DW, Okitsu SL, et al. Evidence of blood stage efficacy with a virosomal malaria vaccine in a phase IIa clinical trial. PLoS ONE 2008;3:e1493.

29. Duncan CJA SS, Ewer K, Douglas AD, Collins KA, Halstead FD, Elias SC, Lillie PJ, Rausch K, Aebig J, Miura K, Edwards NJ, Poulton ID, Hunt-Cooke A, Porter DW, Thompson FM, Rowland R, Draper SJ, Gilbert SC, Fay MP, Long CA, Zhu D, Wu Y, Martin LB,. Impact on Malaria Parasite Multiplication Rates in Infected Volunteers of the Protein-in-Adjuvant Vaccine AMA1-C1/Alhydrogel + CPG 7909. PLoS ONE 2011;In press

30. Sanderson F, Andrews L, Douglas AD, Hunt-Cooke A, Bejon P, Hill AV. Blood-stage challenge for malaria vaccine efficacy trials: a pilot study with discussion of safety and potential value. Am J Trop Med Hyg 2008;78:878-83.

31. Cheng Q, Lawrence G, Reed C, et al. Measurement of Plasmodium falciparum growth rates in vivo: a test of malaria vaccines. Am J Trop Med Hyg 1997;57:495-500.

32. Pombo DJ, Lawrence G, Hirunpetcharat C, et al. Immunity to malaria after administration of ultra-low doses of red cells infected with Plasmodium falciparum. Lancet 2002;360:610-7.

33. Heppner DG, Gordon DM, Gross M, et al. Safety, immunogenicity, and efficacy of Plasmodium falciparum repeatless circumsporozoite protein vaccine encapsulated in liposomes. J Infect Dis 1996;174:361-6.

34. Duncan CJA SS, Ewer K, Douglas AD, Collins KA, Halstead FD, Elias SC, Lillie PJ, Rausch K, Aebig J, Miura K, Edwards NJ, Poulton ID, Hunt-Cooke A, Porter DW, Thompson FM, Rowland R, Draper SJ, Gilbert SC, Fay MP, Long CA, Zhu D, Wu Y, Martin LB, Anderson CF, Lawrie AM, Hill AVS, Ellis RD. Impact on Malaria Parasite Multiplication Rates in Infected Volunteers of the Protein-in-Adjuvant Vaccine AMA1-C1/Alhydrogel + CPG 7909. PLoS ONE 2011;In press.

35. Rickman LS, Jones TR, Long GW, et al. Plasmodium falciparum-infected Anopheles stephensi inconsistently transmit malaria to humans. Am J Trop Med Hyg 1990;43:441-5.

36. Ploemen IH, Prudencio M, Douradinha BG, et al. Visualisation and quantitative analysis of the rodent malaria liver stage by real time imaging. PLoS ONE 2009;4:e7881.

37. Hoffman SL, Billingsley PF, James E, et al. Development of a metabolically active, non-replicating sporozoite vaccine to prevent Plasmodium falciparum malaria. Hum Vaccin 2010;6:97-106.

38. Lyke KE, Laurens M, Adams M, et al. Plasmodium falciparum malaria challenge by the bite of aseptic Anopheles stephensi mosquitoes: results of a randomized infectivity trial. PLoS ONE 2010;5:e13490.

39. Conroy RM, Pyorala K, Fitzgerald AP, et al. Estimation of ten-year risk of fatal cardiovascular disease in Europe: the SCORE project. Eur Heart J 2003;24:987-1003.

40. FDA. Toxicity Grading Scale for Healthy Adult and Adolescent Volunteers Enrolled in Preventive Vaccine Clinical Trials. 2007.

41. Fortnum H, Lee AJ, Rupnik B, Avery A. Survey to assess public awareness of patient reporting of adverse drug reactions in Great Britain. J Clin Pharm Ther 2011.
